# Droplet-Merging and Dissolution-Induced Intermediate State Strategy Enabled Efficiency > 17.5% for the Printed Organic Solar Cells

**DOI:** 10.1007/s40820-026-02268-8

**Published:** 2026-07-06

**Authors:** Lifeng Sang, Xingze Chen, Chen Chen, Yuanyuan Jiang, Qing Zhang, Ni Yin, Yue Guo, Wei Li, Tao Wang, Xiaozhang Zhu, Qi Chen, Chang-Qi Ma, Qun Luo

**Affiliations:** 1 School of Nano-Tech and Nano-Bionics, University of Science and Technology of China, Hefei, 230027 People’s Republic of China; 2 i-Lab, Suzhou Institute of Nano-Tech and Nano-Bionics, Chinese Academy of Sciences (CAS), Suzhou, 215123 People’s Republic of China; 3 School of Materials Science and Engineering, Wuhan University of Technology, Wuhan, 430070 People’s Republic of China; 4 Beijing National Laboratory for Molecular Sciences, CAS Key Laboratory of Organic Solids, Chinese Academy of Sciences (CAS), Beijing, 100190 People’s Republic of China; 5 Vacuum Interconnected Nanotech Workstation (Nano-X), Suzhou Institute of Nano-Tech and Nano-Bionics, Chinese Academy of Sciences (CAS), Suzhou, 215123 People’s Republic of China

**Keywords:** Organic solar cells, Pre-aggregation, Molecular orientation, Redissolution, Inkjet-printed

## Abstract

Molecular pre-aggregation behavior plays a critical role in the morphology of organic films and the device performance of organic solar cells (OSCs). In the high-boiling-point solvent system, the relatively slow drying crystallization kinetics impacted the solution-state molecular pre-aggregation control, complicating the films crystallization regulation. In this work, a novel approach to regulate the pre-aggregation characteristics of the thin films was developed by constructing a droplet-merging and dissolution-induced intermediate state during inkjet printing (IJP). During this process, three key parameters, the printing temperature, droplet space, and additives, were identified to be critical for the intermediate state formation. This method created an intermediate aggregation state that finally enhanced the crystallization and molecular orientations in films printed from ortho-dichlorobenzene, overcoming the slow kinetics challenge. Consequently, IJP OSCs achieved a record power conversion efficiency of 17.57% through this droplet-merging and dissolution-induced intermediate strategy.

**Supplementary Information:**

The online version contains supplementary material available at 10.1007/s40820-026-02268-8.

## Highlights

- A droplet-merging and dissolution-induced intermediate state strategy was developed to effectively promote molecular crystallization and molecular orientation.
- High performance > 17.5% for small-area devices and high-performance large-area modules were fabricated by inkjet printing through this intermediate state strategy.

**Supplementary Information:**

The online version contains supplementary material available at 10.1007/s40820-026-02268-8.

## Graphical abstract

**Figure:**
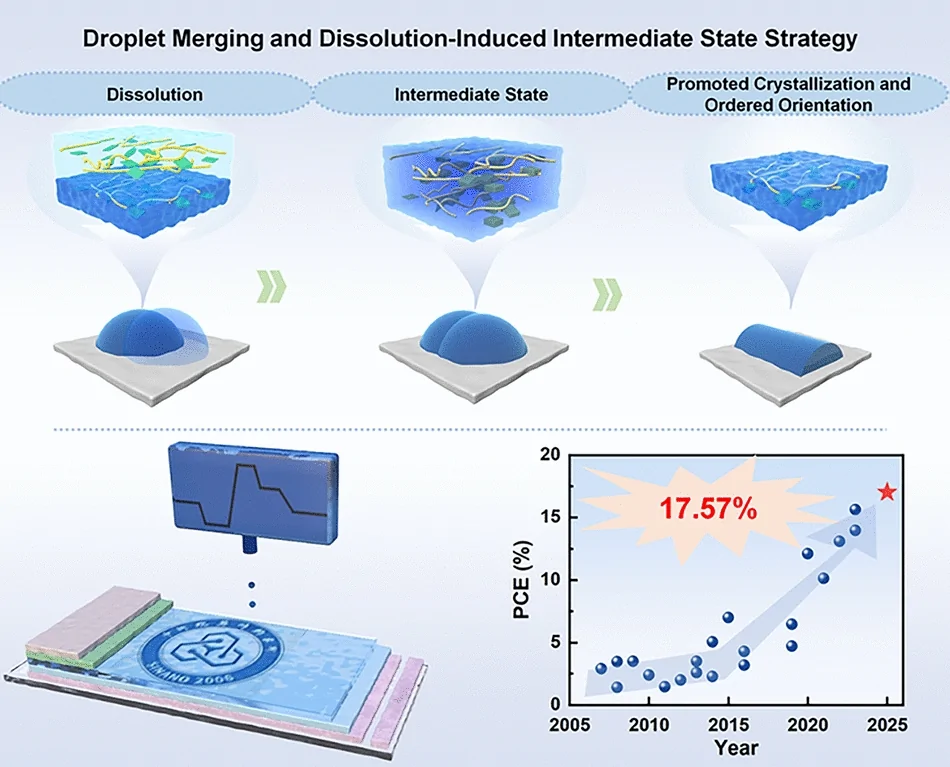

## Introduction

Organic solar cells (OSCs) are an emerging new photovoltaic technology with advantages of low weight [1, 2], flexibility [3–5], and suitability for large-scale solution processing [6–9]. OSCs have great application potential for wearable electronics, indoor photovoltaics, and building-integrated photovoltaics [10, 11]. The efficiency of OSCs has developed rapidly, with power conversion efficiency (PCE) exceeding 21% [12–17].

The morphology of the active layer, especially molecular crystallization and orientation, critically influences the performance of OSCs [18]. Currently, the widely used non-fullerene acceptors (NFAs) are banana-shaped Y-series molecules. These molecules enable multiple packing modes and provide various charge transport pathways, leading to outstanding charge transport properties [19–22]. However, high-efficiency OSCs often rely on low-boiling-point solvents that offer good solubility and fast film-forming kinetics. In the high-boiling-point solvents, although more suitable for large-area processing, the limited solubility would significantly restrict the improvement of device performance. Moreover, the regulation of the film-forming kinetics in high-boiling-point solvent system is more challenging, which hinders the upscaling of OSCs.

Regulating the crystallization behavior in the active layer is crucial. Current strategies can be broadly classified into molecular engineering, additive engineering, and process engineering. In terms of molecular engineering, Chen et al*.* [23] demonstrated that shortening the alkyl side chains promotes solution pre-aggregation, leading to enhanced out-of-plane (OOP) diffraction and increased crystal coherence length, ultimately increasing the fill factor (FF) to 72%. Molecular pre-aggregation capability can also be optimized through fluorine atom positional isomerization via non-covalent intramolecular interactions [24]. Simultaneously, a double-cable conjugated polymer (SC-1F) was proved to be efficient to stabilize the pre-aggregation in solution, which acted as a template for ordered acceptor packing, thereby significantly enhancing the OOP diffraction intensity and enabling device PCE exceeding 20% with a short-circuit current density (*J*_SC_) of 28.86 mA cm^−2^ [25]. Additive engineering plays a critical role as well. Solid additives such as 2-chloro-5-iodopyridine (PDCI) induce crystallization through strong non-covalent interactions with Y-series acceptors, leading to face-on orientation and an improved FF from 70.16 to 78.69% [26]. Besides, oligomers were introduced as the nucleating agents to promote pre-aggregation and accelerate the crystallization kinetics [27]. Liquid additives influence film formation through two mechanisms. First one is direct regulation *via* selective dissolution or intermolecular interactions. For example, DIO promotes face-on orientation, while CN induces edge on orientation [28]. The second mechanism is extending the molecular relaxation time during film formation [29].These mechanisms often work synergistically; for instance, the additive brominated diphenyl ether (DPE-Br) combines a high boiling point with a high dipole moment to optimize vertical phase distribution while forming strong non-covalent interactions with the acceptor L8-BO, enabling device efficiencies exceeding 20% [30]. Process engineering also offers effective control; for example, the hot-substrate method has been shown to suppress the disordered pre-aggregation of donor molecules [31]. Despite these advances, in high-boiling-point solvent systems, the slower drying and crystallization kinetics render conventional solution-state pre-aggregation regulation less effective, warranting further investigation.

In this work, the crystallization kinetics of organic bulk heterojunctions in high-boiling-point solvent ortho-dichlorobenzene (oDCB) were regulated through a dynamic droplet-merging and dissolution strategy. In this strategy, the printing temperature and additives were found to be important for the pre-aggregation intermediate state. A proper pre-aggregation intermediate state was formed through increasing the printing temperature and using BN as the additive, which lead to enhanced molecular crystallization and consistent molecular orientation in the printed films. Ultimately, a record PCE of 17.57% was achieved for the inkjet-printed OSCs, which is significantly higher than the control device of 12.08%. This work demonstrates that constructing an intermediate state through droplet-merging and dissolution uniquely enhances molecular crystallization of the photoactive layer during oDCB fabrication process.

## Experimental Section

### Materials

Poly[(2,6-(4,8-bis(5-(2-ethylhexyl)−3-fluoro)thiophen-2-yl)-benzo[1,2-b:4,5-b']dithiophene))-alt-(5,5-(1',3'-di-2-thienyl-5',7'-bis(2-ethylhexyl)benzo[1',2'-c:4',5'-c']dithiophene-4,8-dione)] (PBDB-T-2F) donor, 2,2'-((2Z,2'Z)-((12,13-bis(2-ethylhexyl)−3,9-diundecyl-12,13-dihydro-[1,2,5]thiadiazolo[3,4-e]thieno[2'',3'':4',5']thieno[2',3':4,5]pyrrolo[3,2-g]thieno[2',3':4,5]thieno[3,2-b]indole-2,10-diyl)bis(methanylylidene))bis(5,6-dichloro-3-oxo-2,3-dihydro-1Hindene-2,1-diylidene))dimalononitrile (BTP-BO-4Cl), 2,2'-((2Z,2'Z)-((12,13-bis(3-ethylheptyl)−3,9-diundecyl-12,13-dihydro-[1,2,5]thiadiazolo[3,4-e]thieno[2'',3'':4',5']thieno[2',3':4,5]pyrrolo[3,2-g]thieno[2',3':4,5]thieno[3,2-b]indole-2,10-diyl)bis(methanylylidene))bis(5,6-difluoro-3-oxo-2,3-dihydro-1H-indene-2,1-diylidene))dimalononitrile (N3), 2,2'-((2Z,2'Z)-((12,13-bis(2-butyloctyl)−3,9-dinonyl-12,13-dihydro-[1,2,5]thiadiazolo[3,4-e]thieno[2'',3'':4',5']thieno[2',3':4,5]pyrrolo[3,2-g]thieno[2',3':4,5]thieno[3,2-b]indole-2,10-diyl)bis(methanylylidene))bis(5,6-dichloro-3-oxo-2,3-dihydro-1H-indene-2,1-diylidene))dimalononitrile (BTP-eC9), and 2,2'-((2Z,2'Z)-((3,9-bis(2-butyloctyl)−12,13-bis(2-butyloctyl)−12,13-dihydro-[1,2,5]thiadiazolo[3,4-e]thieno[2'',3'':4',5']thieno[2',3':4,5]pyrrolo[3,2-g]thieno[2',3':4,5]thieno[3,2-b]indole-2,10-diyl)bis(methanylylidene))bis(5,6-difluoro-3-oxo-2,3-dihydro-1H-indene-2,1-diylidene))dimalononitrile (L8-BO) acceptor was purchased from Hyper, Inc., Jiaxing., and used as received without further purification. Ortho-dichlorobenzene (oDCB) (purity > 99%) and Tetralin (THN) (purity > 99%) were purchased from J&K Scientific Ltd. PEI-Zn was synthesized according to our previous report. The additives 1,8-diiodooctane (DIO), 1-fluoronaphthlene (FN), 1-chloronaphthalene (CN), and 1-Bromonaphthalene (BN) were purchased from TCI (Shanghai) Development Co., Ltd., with purity > 97.0% (GC) and were directly added to the inks as additives.

### Preparation of OSCs

Inverted devices with a device structure of glass/ITO/PEI-Zn/PBDB-T-2F:BTP-BO-4Cl/MoO_*x*_/Al were prepared. First, the ITO glasses were washed sequentially with deionized water and ethanol for 30 min. Before device fabrication, the ITO electrodes were treated with UV ozone for 20 min. Afterward, the ZnO electron transporting layer (ETL) was deposited on the ITO glass through spin-coating at 3000 rpm for 30 s using ZnO ink and then annealed at 150 °C for 10 min. The photoactive layer solution was prepared by dissolving PBDB-T-2F and BTP-BO-4Cl (weight ratio 1:1.2, 13.2 mg mL^−1^) in oDCB and THN composite solvents (volume ratio 9:1) and stirring at 80 °C for 6 h. The organic photoactive layers were deposited through IJP with a Fujifilm Dimatix DMP 2850 inkjet printer, using a printhead with 16 nozzles (individual nozzle diameter of 21 μm, average ink output of around 10 pL per pass). During printing, the applied voltage and meniscus pressure of the print head were set at 30 V and 5 mbar, respectively. The drop spacing was 40 µm, and the substrate temperature ranged from 30 to 45 °C. Finally, the samples were transferred into an evaporation chamber, and 15 nm MoO_x_ and 200 nm Al were thermally evaporated onto the photoactive layer using a shadow mask to obtain OSCs at a vacuum of 6 × 10^−4^ Pa. The donor concentration of the high-concentration ink was 24 mg mL^−1^. During the printing process, the printhead voltage was set at 40 V, the meniscus pressure at 5 mbar, the drop spacing at 80 μm, and the substrate temperature was maintained at 40 °C. The spin-coated device has the same structure as the printed device, with the difference being that the active layer film is spin-coated onto the ETL at a speed of 1000 rpm for 60 s using active layer ink, followed by annealing at 100 °C for 10 min. The structure of the regular device is glass/ITO/PEDOT:PSS/PBDB-T-2F:BTP-BO-4Cl/PDINN/Al. The hole transporting layer (HTL) was prepared by spin-coating PEDOT:PSS ink on the ITO glass at 6000 rpm for 30 s and then annealed at 150 °C for 10 min. The ETL was prepared by spin-coating PDINN ink on the active layer film at 3000 rpm for 30 s. Finally, the samples were transferred into an evaporation chamber, and 200 nm of Al was thermally evaporated onto the active layer through a shadow mask in a vacuum (6 × 10^−4^ Pa) to fabricate OSCs.

### Instruments and Measurements

Current density–voltage (*J-V*) measurements of the OSCs are carried out in a nitrogen glove box using a solar simulator (Zolix, SS150). The external quantum efficiency (EQE) of devices was measured using a homemade EQE measurement system that contained a 150-W tungsten halogen lamp (OSRAM 64610), a monochromator (Zolix, Omni-λ300), a *J–V* converter (Suzhou Dresdner Instruments Ltd.), and a lock-in amplifier (Stanford Research Systems SR 830). Before testing, the instrument was warmed up for 20 min, and the EQE test was calibrated using a standard silicon cell as a reference before sample measurements. Fourier transform photocurrent spectroscopy (FTPS-EQE) measurements were carried out using an integrated system (PECT-600, Enlitech), with a lock-in instrument employed to amplify and modulate the photocurrent. Electroluminescent spectroscopy (*EQE*_EL_) measurements were performed by applying external voltage/current sources to the devices (ELCT-3010, Enlitech). *EQE*_EL_ measurements for all devices were conducted in line with the optimal device preparation conditions.

Grazing-incidence wide-angle X-ray scattering (GIWAXS) was performed using Xenocs Xeuss 3.0 (France) to characterize the molecular buildup of the active layer in the films (test conditions: incidence angle 0.2°, detector distance 130 mm, exposure time 900 s, light source Ga target, wavelength 1.54 Å) at the Vacuum Interconnected Nanotech Workstation (Nano-X) of SINANO. UV–Vis absorption spectra were recorded using a PerkinElmer UV–Vis spectrometer (model Lambda 750). Grazing-incidence small-angle X-ray scattering (GISAXS) was measured by the Ganesha SAXSLAB instrument with an X-ray energy of 8.047 keV (1.54 Å) at TU Munich. The SDD was set to 1045 mm with an incident angle of 0.4° for the collection of the GISAXS data. Transmission electron microscopy (TEM) images were obtained using an FEI Tecnai G2 F20 S-TWIN transmission electron microscope. Photoluminescence excitation (PLE) spectra were measured using an Edinburgh Instruments FLS1000 fluorescence spectrometer. Pre-experiments determined that the acceptor emission peak was located at 930 nm; therefore, the monitored emission wavelength range was set to 850–1150 nm. The excitation wavelength was monitored from 400 to 900 nm with a step size of 1 nm. The excitation slit width was 5 nm, and the emission slit width was 12 nm. All measurements were performed at room temperature.* In situ* UV–Vis absorption measurements were performed using a halogen light source and a general-purpose fast multispectral spectrometer produced by Shaanxi Puguangweishi Technology Co., Ltd., with the optical fiber aligned to focus the light on the center of the film. The sampling interval was 0.5 s.

The molecular electronic properties are calculated by Gaussian 16 with B3LYP/6-31G(d) basis sets, where the long alkyl side chains are simplified to methyl groups to construct the molecular models. The optimized geometries do not show any imaginary frequencies. Estimation of Hansen solubility parameters (HSPs) and Flory–Huggins interaction parameter: The HSPs were estimated according to the functional-group additive method as described by Richter et al*.* [32], and a revised approach based on Hoftyer and Krevelen’s method was employed in this work [33]. The solubility parameters of organic molecules can be calculated by the group-contribution method.

Density functional theory (DFT) calculations were performed on an ORCA5.0.4 software package [34], and molecular dynamics (MD) simulations were conducted via GROMACS 2023 software package with GPU acceleration. The single-point energy of all single molecules was calculated at the B3LYP/def2SVP level, and the atomic charges were fitted using the restrained electrostatic potential (RESP) method by Multiwfn software. The Ztop program was utilized to refine the force field parameters according to the DFT results and produce the topology files of all molecules. An NPT run of 20 ns was performed to obtain the equilibrated configuration after the new boxes were established. The quasi-equilibrium molecular dynamics, in which 100 oDCB molecules were removed after every NPT run of 100 ps, was performed to simulate the solvent evaporation. In all of the MD simulations, the Nosé–Hoover thermostat and C-rescale were employed to control the temperature and pressure of the system, and the velocity-Verlet integrator at a 2.0 fs time step with LINCS algorithm was employed to constrain bonds with hydrogen [35].

## Results and Discussion

### Construction of Intermediate State via Droplet-Merging and Dissolution

**Fig. 1:**
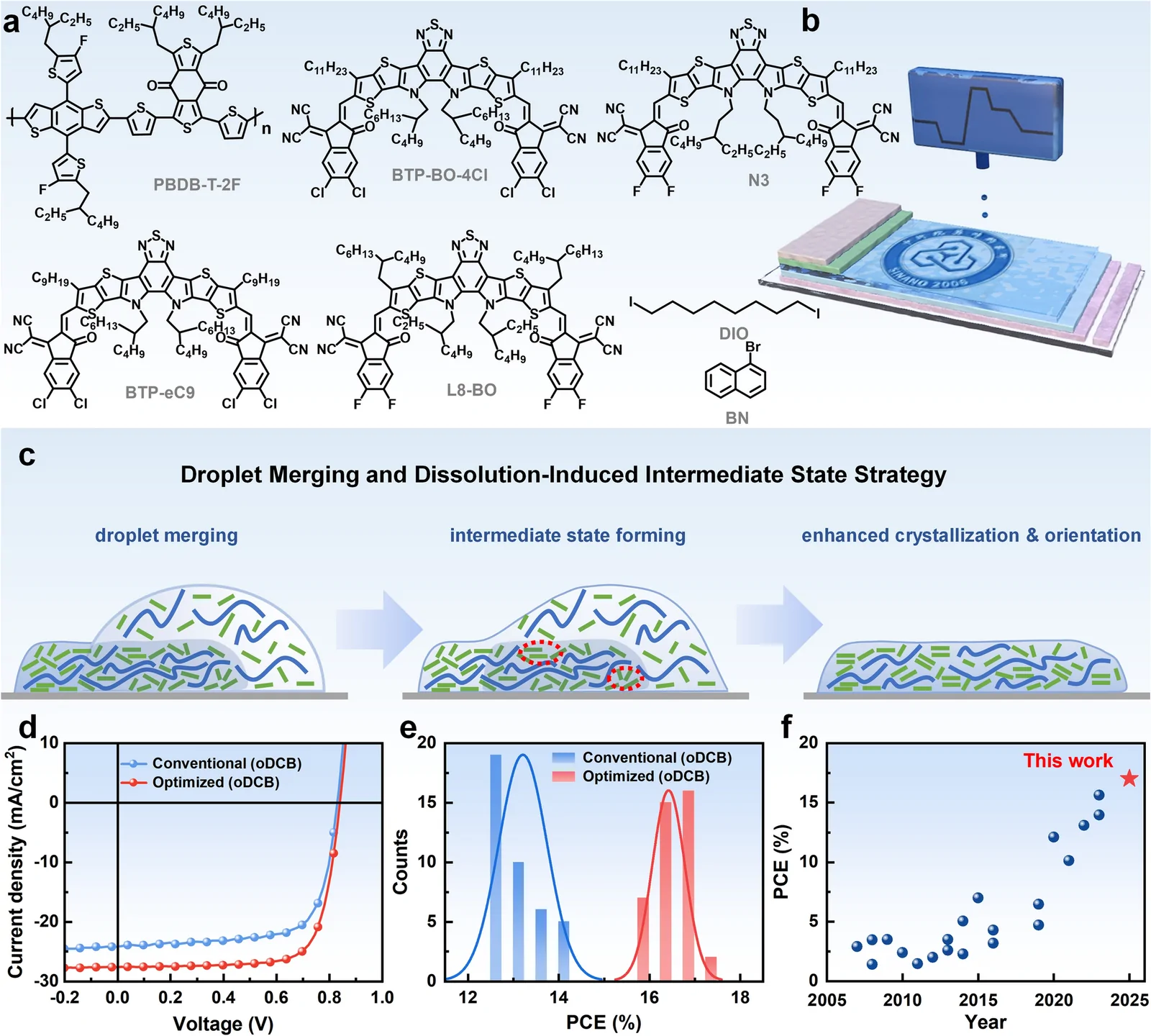
Materials, method, and photovoltaic performance. **a** Structures of polymer, NFAs, and solvent additives used in this work. **b** Schematic of inkjet printing. **c** Schematic diagram of the droplet-merging and dissolution-induced intermediate state strategy. **d** *J–V* curves of devices fabricated through different processing methods. **e** Histogram of PCE of the organic solar cells calculated over 40 devices fabricated through different processing methods. **f** Performance statistics for inkjet-printed devices

The structures of polymer donors, NFAs, and additives used in this work are presented in Fig. 1a. A series of *Y*-series acceptors, *i.e.,* BTP-BO-4Cl, N3, BTP-eC9, and L8-BO, were employed as donors, and 1,8-diiodooctane (DIO) and 1-Bromonaphthalene (BN) were used as the additives. A schematic diagram of the device architecture is shown in Fig. S1, with the structure of glass/ITO/PEI-Zn/PBDB-T-2F:BTP-BO-4Cl/MoO_x_/Al. Before the fabrication of films through IJP, the devices through spin-coating were first fabricated. We know the performance of OSCs is strongly influenced by the solvent and the film deposition method of the organic photoactive layer. When high-boiling-point solvent oDCB is used, the limited solubility and slow film-forming kinetics would lead to poor device performance. As summarized in Table S1, a PCE of 14.43% is achieved for OSCs processed with oDCB, which is much lower than the PCE of the device prepared from a low-boiling-point solvent system chloroform (CF) (16.31%). The grazing-incidence wide-angle X-ray scattering (GIWAXS) measurements revealed a preferential face-on orientation of BTP-BO-4Cl in the blend films from CF solvent (Fig. S2) [36]. In detail, the (010) diffraction peak in the OOP direction at q_z_ = 1.75 Å^−1^ was observed. Such an orientation is beneficial for vertical charge transport [37]. However, in the blend film processed with oDCB solvent, weaker diffraction intensity is observed compared to that from CF solvent system. In addition, a lower proportion of face-on orientation in the blend film is also observed. These morphological features would limit the performance of device.

In this work, a droplet pre-aggregation strategy based on IJP was developed to regulate the crystallization and molecular orientation of films. As shown in the schematic diagram of the IJP process in Figs. 1b and S3, the method is characterized by point-by-point deposition and controllable droplet positioning. This characteristic enables the precise deposition of the active layer films. During IJP, the thickness of film was regulated by controlling the ink concentration and the spacing between adjacent droplets. In addition, since the films are formed through the dynamic dissolution of the former droplet by the latter droplet, the morphology of the IJP films could be modulated through regulating the redissolution process. As shown in Fig. 1c, the schematic of the dynamic redissolution process contains two key steps: (i) deposition of droplets onto the prior printed droplets and (ii) partial redissolution of the former droplet and re-drying. During this process, local gradient temperature and concentration around the droplet significantly influenced the crystallization and molecular stacking. As shown in Fig. 1d, f, the droplet-merging and redissolution regulation strategy enabled device performance of 17.57% prepared from the oDCB, which represents the highest efficiency reported for inkjet-printed OSCs to date [38–45]. In contrast, devices spin-coated with oDCB achieved only 14.43% PCE. This result indicates that the redissolution regulation approach is an effective method to promote the performance of OSCs when preparing through oDCB.

### Intermediate-State-Induced Crystallization and Orientation

**Fig. 2:**
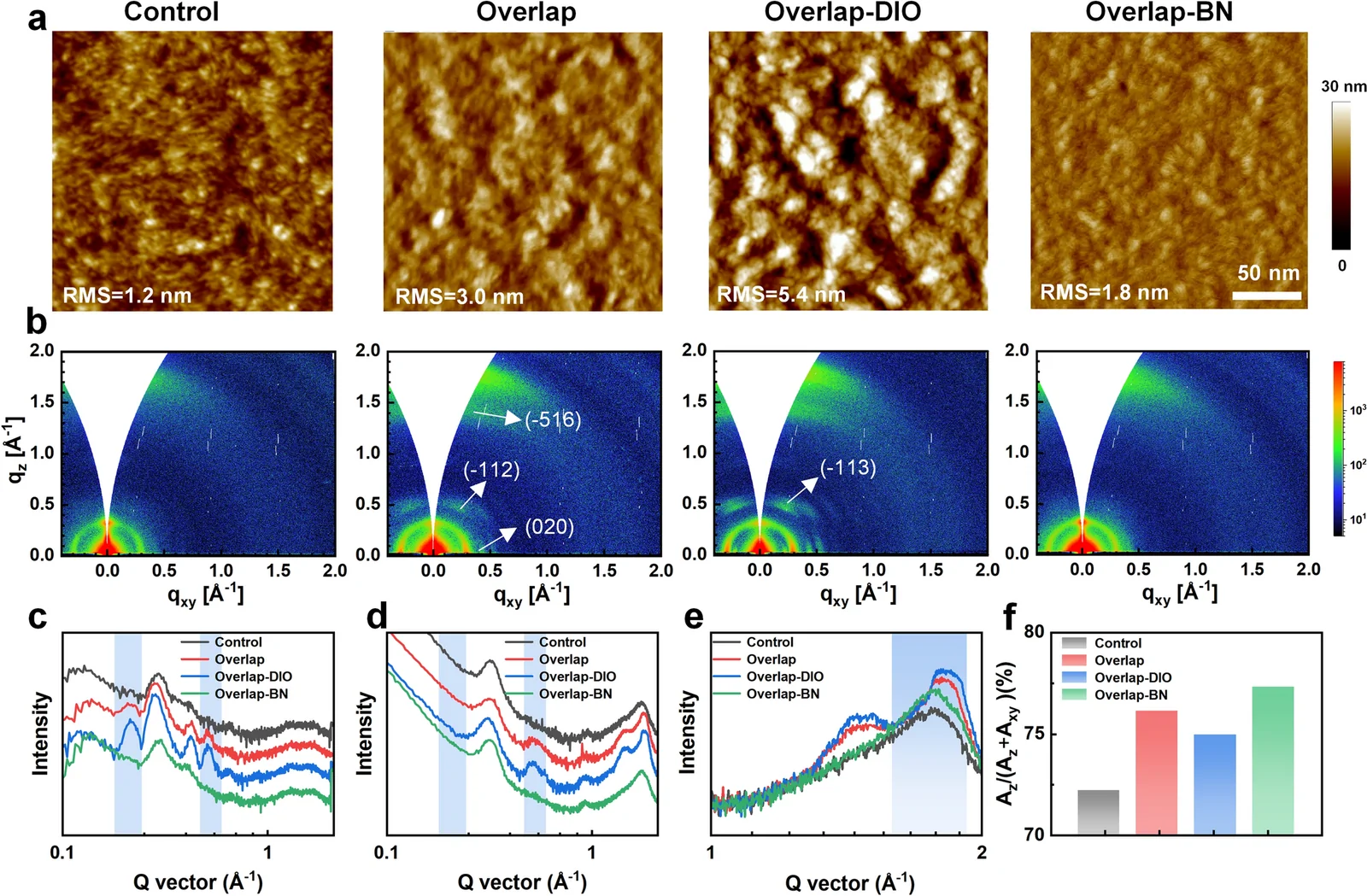
Morphological characterizations of thin films. **a** AFM height images of the printed films processed under Control, Overlap, Overlap-DIO, and Overlap-BN, respectively. **b** 2D GIWAXS patterns of the printed films processed under Control, Overlap, Overlap-DIO, and Overlap-BN, respectively. Corresponding line-cut profiles in the **c** IP and **d** OOP directions. **e** Line-cut profiles in the OOP directions of the (010) peak. **f** Face-on ratio calculated from the pole figure of the (010) peak

The film morphology from different processes was investigated by atomic force microscopy (AFM). As shown in Fig. S4, four inkjet printing conditions were set: (1) Control: The films were printed with a medium droplet spacing to make sure the adjacent droplets just touched without overlapping, representing conventional inkjet printing conditions; (2) Overlap: The droplet spacing was reduced so that adjacent droplets partially overlapped. In the overlapping region, the subsequent deposited ink partially dissolved the previously deposited film, creating a redissolution effect; (3) Overlap-DIO: Using the same droplet spacing as the “Overlap” sample, DIO was added as a liquid additive; (4) Overlap-BN: Using the same droplet spacing as the “Overlap” sample, BN was added as a liquid additive. However, the samples from different processes showed varied microscopic morphologies. As shown in Fig. 2a, for the films deposited from a larger dot spacing, the adjacent droplets were merged to form continuous films with reduced surface roughness. In contrast, film formed through the redissolution process exhibited a higher roughness of 3.0 nm. The use of DIO further increased the roughness to 5.4 nm, while BN reduced the roughness and induced a more distinct fibrous morphology. To clarify the effect of the additives, we compared the AFM height images of neat donor (PBDB-T-2F) and acceptor (BTP-BO-4Cl) films with different additives (Fig. S6). The results show that additives significantly affect the surface morphology of acceptor films, while having a minor effect on donor films, indicating that the additives mainly act on acceptor molecule. However, when spin-coating was utilized to process films with the above additives, a negligible difference was obtained in their AFM images (Fig. S7), indicating that the morphological differences are not solely attributed to the additives.

2D GIWAXS patterns and line-cut profiles of the blend films are shown in Fig. 2b. It showed that the crystallinity of the redissolution films is much stronger than that of the non-redissolution films, which would benefit the higher device performance. However, several unexpected diffraction peaks were observed in these redissolution films. Additionally, we also observed that these extraneous peaks became more pronounced in the films with smaller point spacing, *i.e.,* those subjected to greater remelting (Fig. S8). Based on single-crystal structures and 2D GIWAXS simulations (Fig. S9), these peaks were identified as the (020), (− 113), and (− 516) crystal planes, suggesting that more complex crystallization was formed in the redissolved films [20]. Systematic investigation (Figs. S10 and S11) revealed that droplet-merging during film formation is influenced by the substrate temperature that cooperatively regulates molecular crystallization in printed films. Additives further altered the molecular packing within the films. As shown in Fig. 2b, a newly emerged (− 112) crystal plane was observed in films with the DIO additive. The addition of DIO further intensified diffraction peaks but reduced the proportion of face-on orientation. In contrast, films with BN additive exhibited more uniform crystallization behavior, leading to a preferentially face-on molecular packing configuration with (010) diffraction peak in OOP. It is noteworthy to see that the BN-processed films exhibited enhanced crystallinity and a significantly higher proportion of face-on orientation. This indicates that the molecular crystallization behavior in printed films can be selectively modulated by the additive. As shown in Tables S2 and S3, GIWAXS calculations show π-π stacking distances of 3.56, 3.51, 3.48, and 3.52 Å for Control, Overlap, Overlap-DIO, and Overlap-BN devices, respectively, and CCL values of 12.45, 26.29, 6.84, and 33.56 Å. The merging and dissolution treatment promotes tighter molecular packing, and BN further enhances crystallization, quantitatively confirming enhanced crystallinity and more ordered molecular packing [46]. Enhanced crystallization and orientation facilitate exciton dissociation and charge transport, providing morphological basis for improved *J*_SC_ and FF [47]. However, it was interesting to find that different additives have minimal effects on crystalline orientation during spin-coating (as shown in Fig. S12, all blended films showed face-on dominated crystallization).

The transmission electron microscope (TEM) results are shown in Fig. S13. The Control film exhibits a small donor/acceptor phase separation scale, which is unfavorable for exciton diffusion. With the droplet pre-aggregation strategy, the phase separation size increases in the Overlap film, and the Overlap-DIO film shows large-scale phase separation, which may originate from complex crystallization. In contrast, after Overlap-BN treatment, large-scale phase separation is suppressed, forming a finer interpenetrating network that benefits exciton dissociation and charge transport. Furthermore, grazing-incidence small-angle X-ray scattering (GISAXS) measurements were performed to quantitatively analyze the phase separation behavior of the blend films [48]. The 2D GISAXS patterns, 1D intensity profiles, and detailed analytical data are presented in Fig. S14 and Table S4, respectively. The results reveal that the domain sizes in the Overlap-processed printed films are significantly larger than those in the Control printed films, and these domain sizes further increase in the Overlap-DIO-processed printed films. Notably, the measured large domain size of 58.4 nm exceeds the optimal range for efficient exciton dissociation, and the larger domain size of 88.5 nm generally gives rise to enhanced charge recombination and severe efficiency loss in OSCs [49]. In contrast, the Overlap-BN film exhibits distinctly suppressed phase separation, with domain sizes reduced to 40.5, 21.1, and 9.8 nm, which is favorable for charge transport and extraction. This demonstrates the effectiveness of the droplet pre-aggregation regulation strategy in suppressing molecular aggregation.

Further, different molecular crystallization of the printed films was proved by the ultraviolet–visible (UV–Vis) absorption spectra. As shown in Fig. S15, two characteristic absorption peaks corresponding to the 0–0 and 0–1 transitions were observed in the acceptor absorption range at near 820 and 750 nm, respectively. These peaks are attributed to the aggregated state and the monomeric state, respectively [50]. Notably, the intensity of the 0–1 peak was significantly enhanced in the Overlap films, consistent with the crystallization disorder revealed by GIWAXS. In contrast, the 0–1 peak intensity was markedly reduced in the Overlap-BN films, while DIO resulted in more complex crystallization behavior. These results confirm that BN effectively suppresses the formation of complex crystalline structures. UV–Vis absorption spectra of neat films were further measured. The introduction of DIO further affected the aggregation of BTP-BO-4Cl (Fig. S16). As a comparison, the spin-coated films processed with different additives exhibited nearly identical absorption spectra (Fig. S17), indicating that the formation of the 0–1 peak is impacted by additive structure and the processing method.

Photoluminescence excitation (PLE) spectra were subsequently recorded for films printed under varied conditions. As shown in Fig. S18, the excitation intensity varied with the monitored emission wavelength, reflecting the wavelength-dependent energy transfer between donor and acceptor in the films. For films without redissolution, the excitation peak intensities in the 700–800 and 800–900 nm ranges were comparable, indicating that the two excitation bands produced similar excitation efficiency at the monitored emission wavelength. For films that underwent droplet-merging, the overall excitation intensity was higher than that of the non-redissolved films. In addition, the peak intensity in the 700–800 nm range was notably higher than that in the 800–900 nm range. The change in the intensity ratio between the two excitation bands indicates that the redissolution process differentially affected the excitation associated with these absorption bands.

### Mechanism of Pre-Aggregation Regulated by the Intermediate State

**Fig. 3:**
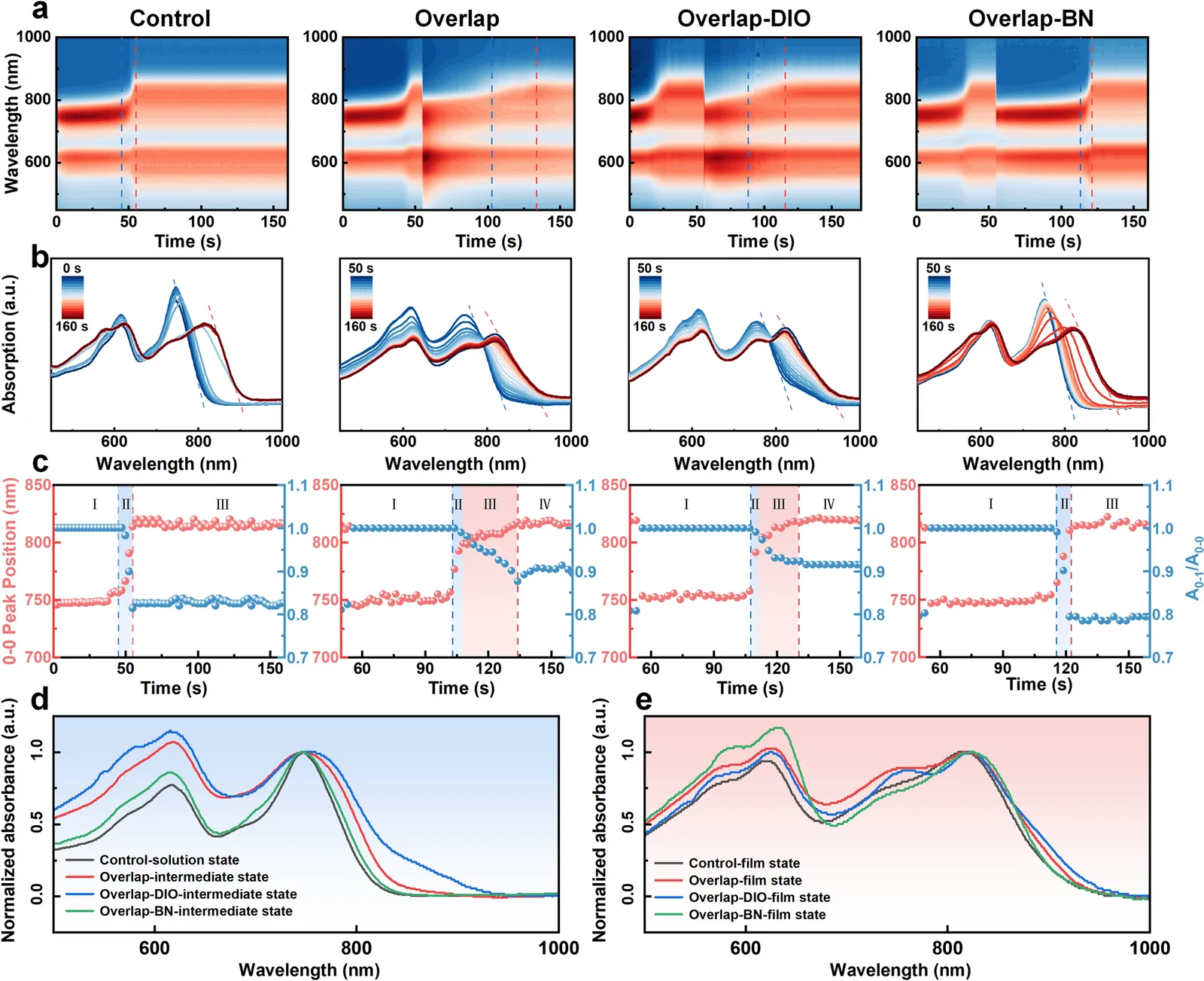
Film-forming kinetics of inkjet-printed PBDB-T-2F:BTP-BO-4Cl films. **a** Time-dependent contour maps of the films processed under Control, Overlap, Overlap-DIO, and Overlap-BN, respectively. **b** Corresponding time-dependent absorption curves. **c** Time evolution of the peak position and the 0–1/0–0 intensity ratio of BTP-BO-4Cl. Absorption spectra of **d** the intermediate state and **e** the final film, extracted from the in situ data

In order to investigate the film-formation kinetics during the dynamic redissolution, *in situ *UV–Vis absorption spectroscopy was employed to monitor the film-formation process (Fig. 3) [51]. The droplet-casting method was used to simulate the IJP process. From the contour plots of the absorption spectra, a gradual redshift in the absorption edge was observed as the solvent evaporated. As shown in Fig. 3, considerable differences in the slopes of the absorption edge were found for those devices, indicating substantial variations in electronic transition energies. In contrast to directly formed films, more compact but disordered packing of NFA molecules was observed in the Overlap and Overlap-DIO films. This result is consistent with the GIWAXS results [52].

To gain direct insight into the aggregation behavior of organic molecules in the films, the evolution of both the acceptor peak positions and the intensity ratio of the 0–1 to 0–0 peaks was extracted from the* in situ* UV–Vis absorption spectra. The absorption peak of BTP-BO-4Cl redshifted from 750 to 820 nm, indicating a transition from solution to film. The process could be described as four steps. Stage I: The former printed droplets were dissolved by the latter droplets. The absorption spectra exhibited no obvious change during this stage. Stage II: Upon the solution concentration exceeding the solubility limit due to solvent evaporation, the organic molecule would nucleate and crystallize, which leads to a rapid redshift in the acceptor absorption peak. Stage III: Heterogeneous nucleation and crystallization of acceptor molecules continue, and excessive aggregation and disordered orientation may sometimes. Stage IV: The formation of the solid film is completed; thus, the absorption peaks of the acceptor are stabilized.

To show the evolution of the molecular aggregates and films crystallization process more clearly, the UV–Vis absorption spectra of the intermediate state were drawn, which is shown in Fig. 3d. In the non-redissolved films, it took approximately 10 s for the direct transition from the solution state to the solid film state. At the critical transition state from solid to solution state, the full width at half maximum (FWHM) of the absorption peak broadened, indicating coexistence of molecular and aggregated states in the intermediate state. Among the three kinds of redissolution films, the DIO additive-involved films showed a much wider FWHM and redshift of the absorption spectra. As a result, the final film showed an obvious typical absorption of the 0–1 peak. This phenomenon was also confirmed by polycrystalline diffraction signals in GIWAXS. Besides, the addition of DIO increased the amount of undissolved pre-crystallization species and resulted in an absorption peak at 880 nm. This also led to more complex molecular crystallization in the film. On the other hand, when BN was used as an additive, the FWHM of the semi-solution was slightly broader, indicating the existence of fewer remaining aggregated molecules. Meanwhile, stage II exhibited a rapid redshift in the acceptor absorption peak, suggesting accelerated crystallization in this process. Figure 3e shows the absorption spectra of the final thin films. The absorption spectrum is consistent with the absorption results of the intermediate state, as shown in Fig. 3d, suggesting that the intermediate state determines the crystallization and aggregates of the printed films. Based on these results, we can see the critical importance of regulating the intermediate redissolution state for the performance of printed OSCs. Specifically, both the films processed with DIO additive and additive-free samples showed a distinct redshift compared to non-redissolution samples, indicating excessive molecular expansion and aggregation within the film layers.

**Fig. 4:**
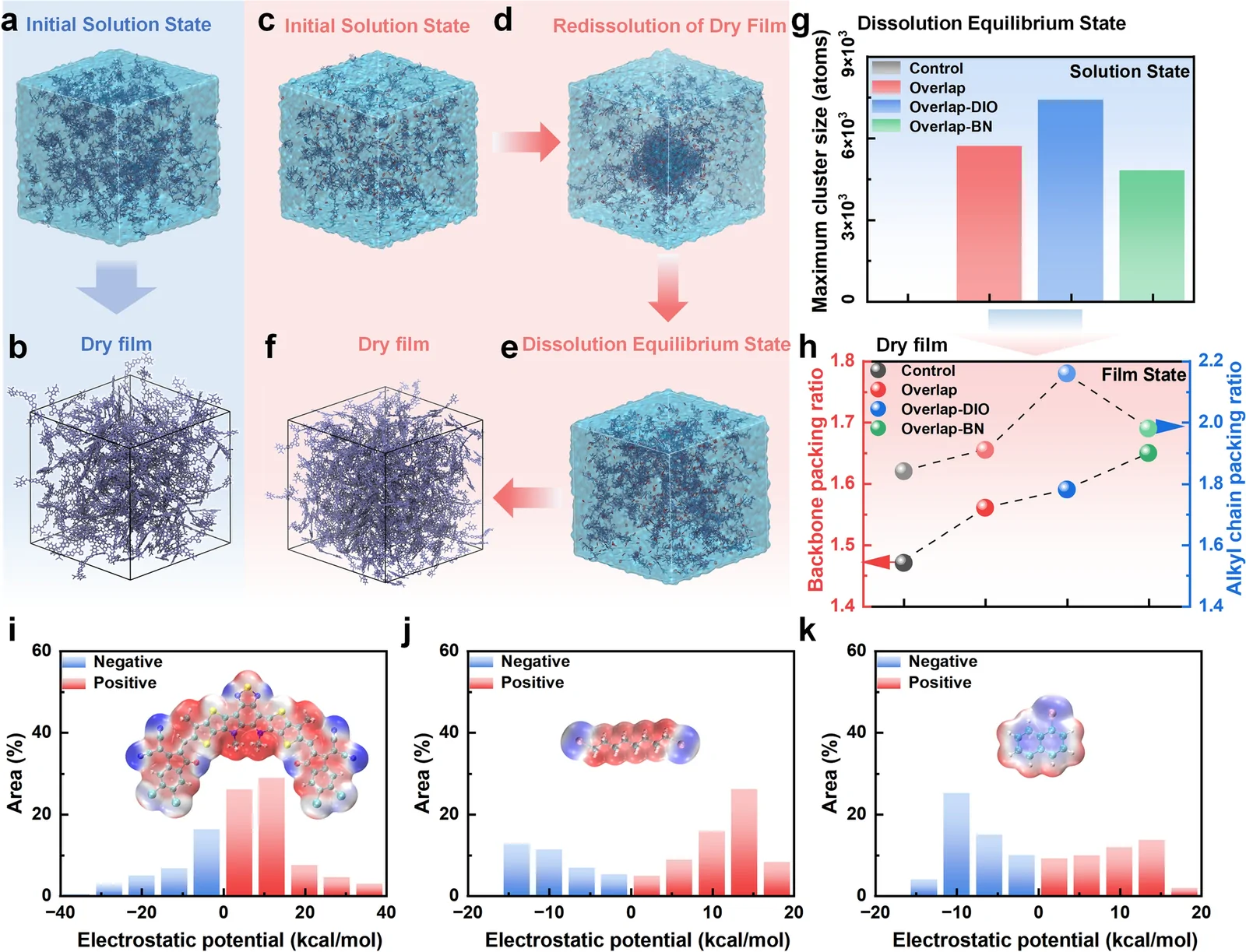
Molecular interactions. Schematic illustrations of molecular dynamics simulations for different film-formation processes: **a** initial solution state, **b** dry film, **c** initial solution state, **d** redissolution of dry film, **e** dissolution equilibrium state, and **f** dry film. **g** Evolution of molecular aggregation cluster size during solvent evaporation. **h** Packing ratios of backbones and alkyl chains in the final film. Molecular electrostatic potential distributions of **i** BTP-BO-4Cl, **j** DIO, and **k** BN

Molecular dynamics simulations (MDSs) were performed to understand the formation mechanism of enhanced molecular crystallization and consistent molecular orientation via the redissolution and additive strategies (Figs. 4 and S19). As shown in Fig. 4a, b, a concentrated ink was modeled by placing 120,000 oDCB molecules and 400 NFA molecules in a rectangular box, where NFAs aggregate upon the evaporation of oDCB. After complete solvent evaporation, an NFA film was obtained. The packing ratios of the backbone and alkyl chains were calculated as the number of stacked units divided by the corresponding atom counts. Detailed packing parameters are summarized in Table S5. In the directly formed NFA film (Fig. 4b), the backbone packing ratio was 1.47, and the alkyl chain packing ratio was 1.84. As shown in Fig. 4c–f, for the film involving redissolution, a system of 60,000 oDCB molecules and 200 NFAs was first evaporated until 99% of the solvent was removed. The concentrated system was then transferred to a new box containing 60,000 oDCB molecules and 200 NFAs. A second evaporation step was set to simulate the film redissolution and re-deposition. As shown in Fig. 4d, the incomplete dissolute films might act as nucleation precursors, lowering the nucleation barrier and thereby enhancing crystallization. During re-evaporation, the largest NFA cluster contained about 5,700 atoms, with the backbone packing ratio and the alkyl chain packing ratio increased to 1.56 and 1.91, respectively (Fig. 4h). This indicates that the redissolution and re-deposition process enhanced molecular crystallization within the film. When additives were further included in the simulation, in systems with additives, larger NFA aggregates were clearly observed during solvent re-evaporation for the DIO-based system (largest cluster > 7,400 atoms), while the BN-based system showed a smaller maximum cluster of only 4,800 atoms (Fig. 4g), suggesting the hindered over-aggregation enabled by BN. After complete evaporation of solvents and additives, dense NFA films were obtained. Among them, the BN-assisted film exhibited relatively balanced backbone and alkyl chain packing, whereas the DIO-assisted film showed significantly increased alkyl chain packing, indicating that the over-aggregation of NFAs might be caused by the increased alkyl chain packing enabled by DIO, as demonstrated in previous work [53, 54].

The Flory–Huggins interaction parameters (*χ*) between the acceptor and additives were calculated through the group-contribution method. The parameters were evaluated based on intermolecular interactions, including dispersion force (δ_D_), polar force (δ_P_), and hydrogen bonding capability (δ_H_) [32]. Specific results are shown in Fig. S20**.** The interaction parameters between various additives and organic molecules, along with their boiling points, are listed in Table S6. A higher *χ* value was observed between oDCB and BTP-BO-4Cl compared to that with PBDB-T-2F, indicating limited solubility of the solvent for the NFA. As a result, during redissolution, molecular aggregates could not be fully dissolved, leading to enhanced crystallization in the film. The *χ* value between DIO and BTP-BO-4Cl was even higher, indicating poorer compatibility and resulting in more complex crystallization after redissolution. In contrast, the *χ* value between BN and BTP-BO-4Cl was 0.25, suggesting good compatibility and enabling more complete dissolution of the film, ultimately leading to enhanced ordered crystallization. As shown in Fig. 4i, k, theoretical simulations based on density functional theory (DFT) revealed that the backbones of *Y*-series NFAs exhibit positive electrostatic potential (ESP) values. The DIO additive also showed a positive ESP at its molecular center. The minimal difference in ESP between DIO and the NFA backbone resulted in poor compatibility. In contrast, the BN additive exhibited negative ESP centers, which enhanced intermolecular electrostatic interactions with BTP-BO-4Cl [55, 56]. This promoted more ordered crystallization of the NFA.

### Photovoltaic Performance of Inkjet-Printed Devices

**Fig. 5:**
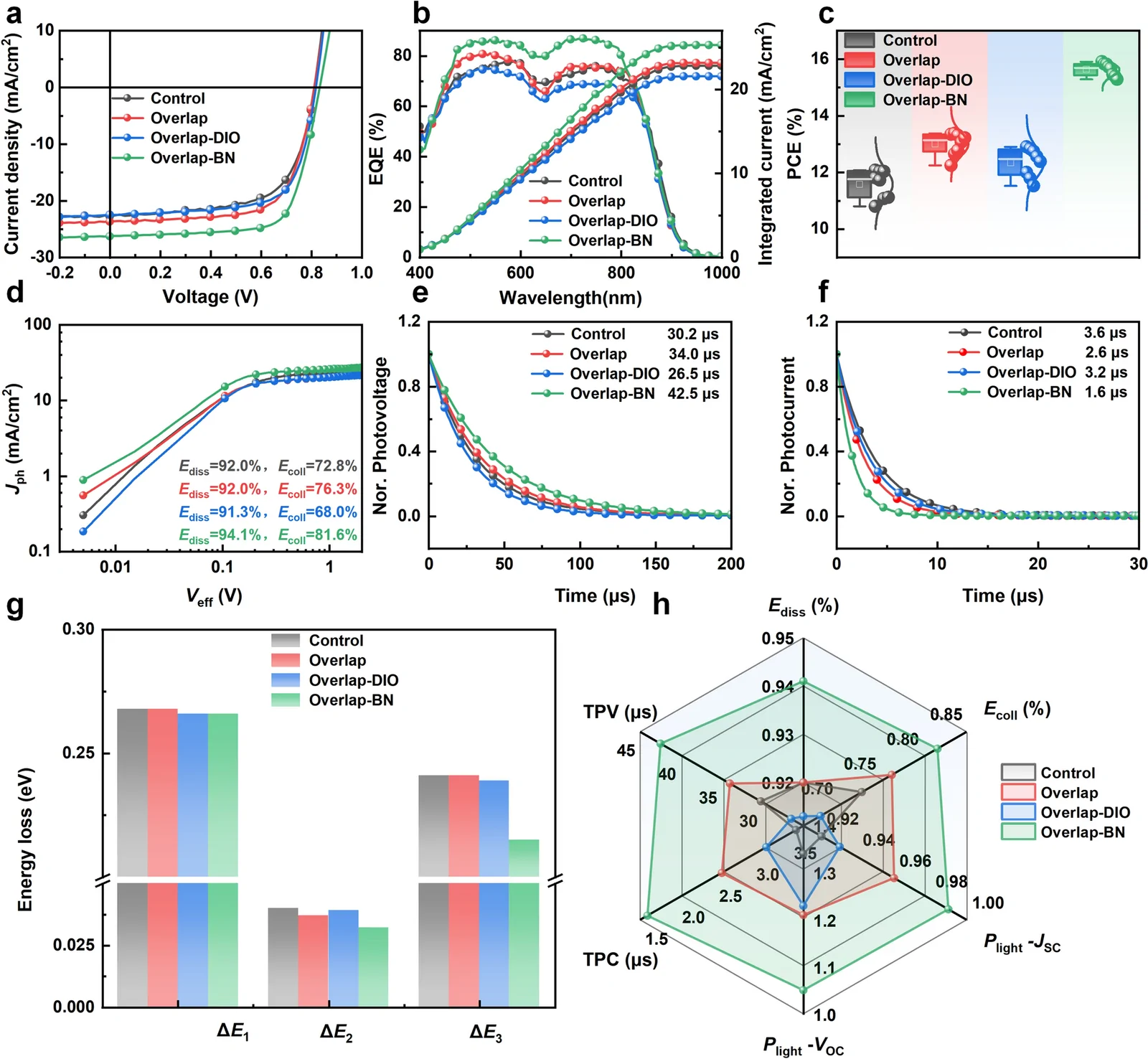
Photovoltaic properties of the inkjet-printed OSCs-based PBDB-T-2F:BTP-BO-4Cl fabricated through different processing methods. **a** *J–V* curves, **b** corresponding EQE spectra, **c** PCE statistic, **d** *J*_ph_ versus *V*_eff_ curves, **e** transient photovoltage (TPV), **f** transient photocurrent (TPC), **g** energy loss analysis diagram, and **h** comparative graph of the parameters of PBDB-T-2F:BTP-BO-4Cl fabricated under Control, Overlap, Overlap-DIO, and Overlap-BN, respectively

Based on optimized morphology, a series of OSCs were fabricated and optimization data are provided in Table S7. Figure 5a presents the current density–voltage (*J–V*) characteristics of the best-performing devices under different conditions, and the corresponding photovoltaic parameters are summarized in Table 1. For the device without droplet redissolution effect, a PCE of 12.08% was achieved, with an open-circuit voltage (*V*_OC_) of 0.813 V, *J*_SC_ of 22.70 mA cm^−2^, and FF of 65.37%. In contrast, printed devices fabricated with lower concentration and smaller droplet spacing exhibited a *V*_OC_ of 0.814 V, a *J*_SC_ of 23.80 mA cm^−2^, an FF of 68.83%, and a PCE of 13.34%. This indicates that the redissolution process can improve the *J*_SC_ and FF of the devices. However, raising the processing temperature induced a loss in *J*_SC_ (Fig. S21). This was further confirmed when the incompatible additive DIO was used; *J*_SC_ sharply decreased to 22.62 mA cm^−2^. Conversely, introducing good-solvent additives BN in the solution, the *J*_SC_ significantly increased to 26.39 mA cm^−2^. Consequently, a *V*_OC_ of 0.836 V, a *J*_SC_ of 26.39 mA cm^−2^, an FF of 72.08%, and a PCE of 15.90% were obtained for the BN-involved devices. The external quantum efficiency (EQE) spectra (Fig. 5b) showed EQE-integrated current of (*J*_SC-cal_) 22.82, 23.16, 21.55, and 25.28 mA cm^−2^ for the Control, Overlap, Overlap-DIO, and Overlap-BN devices, respectively. This result is consistent with the value from* J* to *V* curves.

**Table 1:** Photovoltaic parameters of IJP OSCs, fabricated through different processing methods, under AM 1.5 G 100 mW cm^−2^ illumination

Condition^a^	*V*_OC_ (V)	*J*_SC_ (mA cm^−2^)	FF (%)	PCE (%)
Control	0.813	22.70	65.37	12.08
	0.805 ± 0.002	23.04 ± 0.36	62.45 ± 2.56	11.59 ± 0.51
Overlap	0.814	23.80	68.83	13.34
	0.810 ± 0.005	23.24 ± 0.49	69.03 ± 0.71	13.00 ± 0.40
Overlap-DIO	0.820	22.62	69.58	12.90
	0.816 ± 0.004	21.51 ± 0.91	70.12 ± 0.70	12.30 ± 0.55
Overlap-BN	0.836	26.39	72.08	15.90
	0.833 ± 0.002	26.17 ± 0.25	71.72 ± 0.65	15.63 ± 0.23
Overlap-FN	0.830	24.50	71.92	14.62
	0.822 ± 0.004	24.24 ± 0.45	70.67 ± 0.72	14.08 ± 0.28
Overlap-CN	0.836	25.67	71.27	15.30
	0.831 ± 0.006	25.20 ± 0.63	71.03 ± 1.30	14.87 ± 0.32
Overlap-BN (Ternary)	0.843	27.72	75.22	17.57
	0.839 ± 0.004	27.72 ± 0.18	73.99 ± 0.85	17.20 ± 0.24

^a^Average device performance parameters are calculated on eight individual devices

The exciton dissociation and carrier recombination characteristics in different devices were further examined. Initially, the relationship between the photocurrent density (*J*_ph_) and the effective voltage (*V*_eff_) was analyzed to quantify charge-generation efficiency (Fig. 5d). Here, *J*_ph_ was defined as *J*_L_ minus *J*_D_, where *J*_L_ and* J*_D_ denote the current densities measured under illumination and in the dark, respectively. *V*_eff_ was calculated as* V*_0_ minus *V*_a_, where* V*_0_ was the voltage when *J*_ph_ was zero, and *V*_a_ was the applied bias voltage. The standard control device exhibited an exciton dissociation efficiency (*E*_diss_) of 92.0% and an exciton collection efficiency (*E*_coll_) of 72.8%. In contrast, the device with optimized redissolution treatment showed an improved *E*_coll_ of 76.3%, which is attributed to enhanced crystallization. When DIO was employed as an additive, *E*_diss_ dropped to 91.3%, and *E*_coll_ to 68.0%, consistent with the impact of excessive aggregation on *J*_SC_. Upon incorporation of BN as an additive, the device achieved an *E*_diss_ of 94.1% and *E*_coll_ of 81.6%, demonstrating highly efficient exciton dissociation and collection, leading to significantly improved *J*_SC_.

As shown in Fig. 5e, f, transient photovoltage (TPV) and transient photocurrent (TPC) measurements were conducted to evaluate carrier recombination and carrier extraction dynamics in the devices. Carrier-lifetime values extracted from TPV for Control, Overlap, Overlap-DIO, and Overlap-BN samples were 30.2, 34.0, 26.5, and 42.5 ms, respectively. The corresponding carrier extraction times from TPC were 3.6, 2.6, 3.2, and 1.6 ms, respectively. These results indicate that controlled enhancement of crystallization effectively suppresses carrier recombination. Importantly, the rapid photocurrent decay observed for devices with good-solvent additives confirms efficient charge extraction. The dependence of device performance on incident light intensity *P*_light_ is closely related to the recombination process. To elucidate the carrier recombination behavior, the light-intensity-dependent *J*_SC_ and *V*_OC_ for representative devices were analyzed; the corresponding results are presented in Fig. S22. The values for Control, Overlap, Overlap-DIO, and Overlap-BN devices were 0.92/1.34, 0.96/1.21, 0.93/1.23, and 0.99/1.05, respectively. These results indicate that BN simultaneously suppresses bimolecular and monomolecular recombination, thereby enhancing *J*_SC_ and FF. Overall, these results demonstrate that promoting backbone packing and controlling molecular orientation enable efficient charge extraction and recombination suppression, thereby enhancing photocurrent generation.

To elucidate the origin of the enhanced *V*_OC_ in OSCs, the total energy loss (*E*_loss_) was systematically quantified. Within the Shockley–Quizzer (SQ) framework, *E*_loss_ was decomposed into three components: *E*_loss_ = Δ*E*_1_ + Δ*E*_2_ + Δ*E*_3_ [57]. The detailed *E*_loss_ parameters for each part are summarized in Table 2 and Figs. 4, S23, and S24. Δ*E*_1_, determined by the bandgap of the blended film, accounts for unavoidable radiative recombination losses above the bandgap. Δ*E*_2_ refers to radiative recombination losses below the bandgap, attributed to absorption by low-energy charge-transfer (CT) states. Δ*E*_2_ can be reduced by optimizing the CT-state energy, decreasing energetic disorder, or reducing the reorganization energy [58–60]. The Δ*E*_1_ and Δ*E*_2_ values were comparable for all the devices. Δ*E*_3_ originated predominantly from strong non-radiative recombination of CT states and charges and was sensitive to interfacial morphology [61–64]. Notably, as shown in Fig. 5g, BN-possessed devices had the smallest Δ*E*_3_ of 0.215 eV, which is attributed to its higher electroluminescence external quantum efficiency (EQE_EL_). This reduction was ascribed to strong π-π interactions that suppressed non-radiative energy loss caused by vibrations of C–C and C–H bonds in the alkyl chains [65]. In contrast, devices exhibiting more disordered molecular packing displayed lower EQE_EL_ and larger Δ*E*_3_. Consequently, the device containing BN exhibited the lowest total *E*_loss_ of 0.513 eV, while Control, Overlap, and Overlap-DIO devices had higher *E*_loss_ values of 0.549, 0.546, and 0.544 eV, respectively. The reduced Δ*E*_3_ and *E*_loss_ in Overlap-BN devices were linked to efficient ground-state charge generation, which occupies trap states and suppresses trap-assisted recombination. Thus, BN reduces voltage loss and enhances *V*_OC_. Moreover, as shown in Fig. S25, energetic disorder was quantified by fitting the FTPS-EQE spectra with the Urbach model [66–69]. Overlap-BN devices showed the smallest Urbach energy (*E*_u_) of 24.41 meV. In contrast, Control, Overlap, and Overlap-DIO devices exhibited larger *E*_u_ values of 26.05, 25.89, and 25.73 meV, respectively. The reduced energetic disorder, facilitated by enhanced backbone packing and face-to-face molecular stacking, improved film homogeneity and narrowed the CT-state distribution [70], thereby increasing the FF and PCE [71].

**Table 2:** Detailed *E*_loss_ of the PBDB-T-2F:BTP-BO-4Cl devices fabricated through different processing methods

Condition	*E*_g_^pv^ (eV)	*V*_OC_^mea^ (V)	q*V*_OC_^sq^ (eV)	q*V*_OC_^rad^ (eV)	*E*_loss_ (eV)	Δ*E*_1_ (eV)	Δ*E*_2_ (eV)	Δ*E*_3_ (eV)	EQE_EL_ (× 10^−4^)
Control	1.429	0.813	1.161	1.121	0.549	0.268	0.040	0.241	0.897
Overlap	1.430	0.814	1.162	1.125	0.546	0.268	0.037	0.241	1.169
Overlap-DIO	1.408	0.820	1.142	1.103	0.544	0.266	0.039	0.239	1.542
Overlap-BN	1.407	0.836	1.141	1.109	0.513	0.266	0.032	0.215	1.997

Figure S26 presents the charge-carrier density (*n*_CE_) versus *V*_OC_ characteristics for devices printed under various conditions. The Overlap-BN devices exhibited the highest *n*_CE_, indicating the most efficient charge generation, which is conducive to achieving a high *J*_SC_. It also displayed a longer carrier lifetime (τ), which facilitated charge transport and collection and consequently improved device performance. This observation was corroborated by the recombination coefficient k. Compared to Control, Overlap, and Overlap-DIO devices, the Overlap-BN devices exhibited lower k values across all measured charge densities, signifying suppressed recombination and efficient charge transport.

For comparison, devices containing various additives were fabricated by spin-coating from oDCB. The corresponding *J–V* characteristics are presented in Fig. S27, and the extracted photovoltaic parameters are summarized in Table S8. Interestingly, all the spin-coated devices exhibited comparable performance, indicating similar exciton dissociation, recombination, and extraction dynamics. This result agreed with the identical molecular orientations of the spin-coated films, whatever kinds of additives were used. This may be because spin-coating inherently involves rapid film formation. The evaporation and drying rate difference between higher-boiling-point additives and the high-boiling-point solvent is minimal, making it difficult to induce significant phase separation or molecular orientation changes in the active layer during the spin-coating process. In contrast, redissolution during inkjet-printed active layer deposition enhanced molecular crystallization. The subsequent use of additives enabled selective control over crystallization and intermolecular interactions. This resulted in tailored active layer morphology and improved device efficiency in OSCs processed with dynamic re-solubilization.

### Universality of the Droplet-Merging and Dissolution Strategy

Besides the BTP-BO-4Cl organic system, a similar phenomenon was observed in other *Y*-series binary systems, such as PBDB-T-2F:N3, PBDB-T-2F:BTP-eC9, and PBDB-T-2F:L8-BO. As shown in Figs. S28–S30, overlap films exhibit more complex crystallization behavior compared to non-redissolved films. Crystalline disorder was exacerbated by DIO addition, while molecular ordering in printed films was optimized by BN. This trend, which is consistent across multiple systems, resembles that in the PBDB-T-2F:BTP-BO-4Cl system. Furthermore, the proportion of face-to-face orientation was significantly increased by the BN additive. Moreover, the UV–Vis absorption spectra of other material systems, such as PBDB-T-2F:BTP-eC9 and PBDB-T-2F:L8-BO systems (Fig. S31), also showed absorption peaks at 0–1 peaks. This demonstrates the universal effect of redissolution or DIO additive in enhancing the relative intensity of the 0–1 absorption peak.

As shown in Figs. S32 and S33, similar characteristics were observed in the dynamic film-formation processes of the PBDB-T-2F:BTP-eC9 and PBDB-T-2F:L8-BO systems. When DIO was used as an additive, incomplete dissolution of the primary film led to the presence of large pre-aggregated acceptor in the blend solution, resulting in complex crystallization behavior in the films. In contrast, when BN was used as the additive, the pre-aggregated acceptor was much smaller, which acted as the crystallization nucleating agent, and led to a rapid and ordered molecular crystallization. This indicates that the crystallization of *Y*-series NFAs can be selectively modulated by constructing a semi-solid intermediate state through droplet redissolution.

**Fig. 6:**
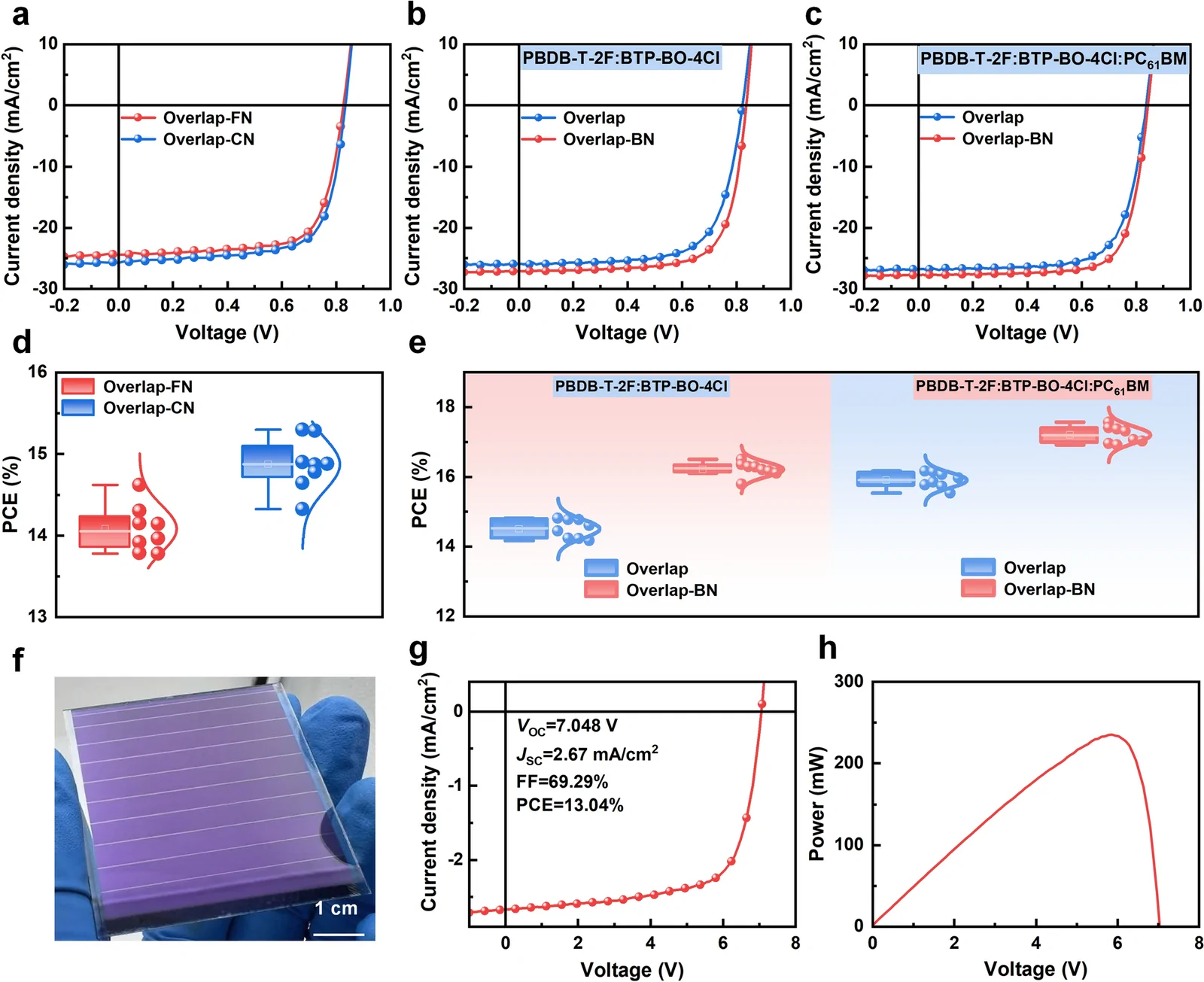
Universality and module device. **a** *J–V* curves of PBDB-T-2F:BTP-BO-4Cl fabricated with different additives. *J–V* curves of the conventional **b** binary and **c** ternary OSCs. **d** and **e** PCE distribution under various conditions. **f** Photograph, **g** *J–V* curves, and **h** *P–V* curves of the module

The intermediate state regulation strategy was further extended to 1-fluoronaphthlene (FN) and 1-chloronaphthalene (CN) additives. Enhanced and ordered crystallization was observed in overlap-processed films (Fig. S34). Good miscibility between the NFA and FN/CN was confirmed, along with strong electrostatic interactions (Table S9 and Fig. S35). By utilizing additives, a larger fraction of acceptor molecules was retained in solution during the redissolution stage. Molecular pre-aggregation was better controlled, resulting in enhanced bulk crystallinity and a preferred face-on orientation [72]. The device performance was improved correspondingly. As shown in Fig. 6a, the CN- and FN-based devices gave performance parameters of 0.830 and 0.836 V for *V*_OC_, 24.50 and 25.67 mA cm^−2^ for *J*_SC_, 71.92% and 71.27% for FF, and 14.62% and 15.30% for PCE, respectively. We find that when different additives (FN, BN, and CN) are used, there are certain differences in device performance. This would be due to the different molecular polarity and electrostatic potential distributions of FN, BN, and CN. Theoretical calculations show that BN exhibits the strongest electrostatic interaction with the acceptor and the best compatibility with both donor and acceptor, thus showing the most significant regulation of molecular aggregation behavior. GIWAXS results show that BN-treated films exhibit the highest (010) peak intensity and the largest crystallite coherence length, indicating the strongest promotion of acceptor crystallinity and face-on orientation. AFM shows that BN-treated films have the lowest roughness and optimal phase separation scale, which improves interfacial contact and charge transport (Fig. S36). Based on the droplet-merging and dissolution regulation strategy proposed in this work, additives with the following characteristics may also be effective: (1) functional groups with strong interactions with acceptor molecules, (2) good compatibility with organic molecules, and (3) conjugated structures similar to organic donor or acceptor molecules.

The additive strategy was further extended to conventional OSCs. The control device based on PBDB-T-2F:BTP-BO-4Cl exhibited a *V*_OC_ of 0.822 V, a *J*_SC_ of 25.91 mA cm^−2^, an FF of 69.56%, and a PCE of approximately 14.48% (Fig. 6b, c, Table S10). Upon incorporation of BN as an additive, *J*_SC_ was increased markedly to 27.10 mA cm^−2^, yielding an improved PCE of 16.50%. Similarly, the ternary control device comprising PBDB-T-2F:BTP-BO-4Cl:PC_61_BM exhibited a *V*_OC_ of 0.836 V, a *J*_SC_ of 26.79 mA cm^−2^, an FF of 72.19%, and a PCE of 16.16%. After BN incorporation, *J*_SC_ rose significantly to 27.72 mA cm^−2^, and the PCE reached 17.57%. Notably, both binary and ternary OSCs outperformed previously compared to the reported inkjet-printed OSCs, demonstrating the broad applicability of the additive strategy.

Building on the above findings, large-area organic photovoltaic modules were fabricated by IJP. A schematic diagram of the module structure is shown in Fig. S37, which is consistent with the single-junction device structure. A representative 24 cm^2^ module, as illustrated in Fig. 6f, showed the inkjet-printed module delivered a PCE of 13.04%, along with a *V*_OC_ of 7.048 V, a *J*_SC_ of 2.67 mA cm^−2^, and an FF of 69.29%. Detailed photovoltaic parameters for the OSCs are listed in Table S11. The successful fabrication of the module underscores the practical potential of the IJP for OSCs.

## Conclusions

In this work, we developed a strategy to regulate the pre-aggregation of molecules in the printed film, which effectively promoted crystallization and enabled molecular orientation consistency. By leveraging the unique feature of inkjet printing that allows microdroplet redissolution of the previous droplet, we effectively induced an intermediate state by adjusting temperature, redissolution spacing, and additives during printing. Ultimately, inkjet-printed OSCs achieved a record PCE of 17.57%. This work provided a new method to regulate the pre-aggregation of molecules in the semi-solid state, which effectively regulated the crystallization and orientation, and finally promoted the performance of the printed devices.

## Supplementary Information

Below is the link to the electronic supplementary material.

Supplementary file1 (DOCX 6534 kb)

## References

[1] X. Zheng, Y. Wang, T. Chen, Y. Kong, X. Wu et al., Realizing record efficiencies for ultra-thin organic photovoltaics through step-by-step optimizations of silver nanowire transparent electrodes. FlexMat **1**(3), 221–233 (2024). 10.1002/flm2.30

[2] E. Bihar, D. Corzo, T.C. Hidalgo, D. Rosas-Villalva, K.N. Salama et al., Fully inkjet-printed, ultrathin and conformable organic photovoltaics as power source based on cross-linked PEDOT: PSS electrodes. Adv. Mater. Technol. **5**(8), 2000226 (2020). 10.1002/admt.202000226

[3] Y. Kakei, S. Katayama, S. Lee, M. Takakuwa, K. Furusawa et al., Integration of body-mounted ultrasoft organic solar cell on cyborg insects with intact mobility. NPJ Flex. Electron. **6**, 78 (2022). 10.1038/s41528-022-00207-2

[4] H. Li, J. Le, H. Tan, L. Hu, X. Li et al., Synergistic multimodal energy dissipation enhances certified efficiency of flexible organic photovoltaics beyond 19%. Adv. Mater. **37**(5), 2411989 (2025). 10.1002/adma.202411989 10.1002/adma.202411989 39655352

[5] L. Sun, W. Zeng, C. Xie, L. Hu, X. Dong et al., Flexible all-solution-processed organic solar cells with high-performance nonfullerene active layers. Adv. Mater. **32**(14), 1907840 (2020). 10.1002/adma.201907840 10.1002/adma.201907840 32091160

[6] B. Deng, Y. Li, Z. Lu, K. Zheng, T. Xu et al., The art and science of translucent color organic solar cells. Nat. Commun. **16**, 597 (2025). 10.1038/s41467-025-55924-9 39799128 10.1038/s41467-025-55924-9 PMC11724960

[7] Y. Cui, H. Li, S. Zeng, K. Zhang, H. Wang et al., Boosting uniformity and efficiency of large-area inverted organic photovoltaics *via* ZnO surface energy modulation. Adv. Funct. Mater. **35**(4), 2414317 (2025). 10.1002/adfm.202414317

[8] Y. Cheng, Y. Ji, D. Zhang, X. Liu, Z. Xia et al., Nitrogen-blowing assisted strategy for fabricating large-area organic solar modules with an efficiency of 15.6%. Polymers **16**(11), 1590 (2024). 10.3390/polym16111590 38891536 10.3390/polym16111590 PMC11174350

[9] H. Gu, J. Zhu, H. Chen, G. Zeng, X. Chen et al., Mechanics manipulation in large-area organic solar modules achieving over 16.5 % efficiency. Giant **18**, 100286 (2024). 10.1016/j.giant.2024.100286

[10] Z. Wang, D. Zhang, L. Yang, O. Allam, Y. Gao et al., Mechanically robust and stretchable organic solar cells plasticized by small-molecule acceptors. Science **387**(6732), 381–387 (2025). 10.1126/science.adp9709 39847644 10.1126/science.adp9709

[11] Y. Li, X. Huang, H.K.M. Sheriff Jr., S.R. Forrest, Semitransparent organic photovoltaics for building-integrated photovoltaic applications. Nat. Rev. Mater. **8**(3), 186–201 (2023). 10.1038/s41578-022-00514-0

[12] L. Zhu, M. Zhang, G. Zhou, Z. Wang, W. Zhong et al., Achieving 20.8% organic solar cells *via* additive-assisted layer-by-layer fabrication with bulk p-i-n structure and improved optical management. Joule **8**(11), 3153–3168 (2024). 10.1016/j.joule.2024.08.001

[13] S. Guan, Y. Li, Z. Bi, Y. Lin, Y. Fu et al., Fine-tuning the hierarchical morphology of multi-component organic photovoltaics *via* a dual-additive strategy for 20.5% efficiency. Energy Environ. Sci. **18**(1), 313–321 (2025). 10.1039/D4EE03778B

[14] H. Chen, Y. Huang, R. Zhang, H. Mou, J. Ding et al., Organic solar cells with 20.82% efficiency and high tolerance of active layer thickness through crystallization sequence manipulation. Nat. Mater. **24**(3), 444–453 (2025). 10.1038/s41563-024-02062-0 39824965 10.1038/s41563-024-02062-0

[15] Y. Wang, C. Gao, W. Lei, T. Yang, Z. Liang et al., Achieving 20% toluene-processed binary organic solar cells *via* secondary regulation of donor aggregation in sequential processing. Nano-Micro Lett. **17**(1), 206 (2025). 10.1007/s40820-025-01715-2 10.1007/s40820-025-01715-2 PMC11961838 40167593

[16] S. Xu, Y. Zhang, Y. Sun, P. Cheng, Z. Yao et al., An unprecedented efficiency with approaching 21% enabled by additive-assisted layer-by-layer processing in organic solar cells. Nano-Micro Lett. **17**(1), 37 (2024). 10.1007/s40820-024-01529-8 10.1007/s40820-024-01529-8 PMC11471740 39397182

[17] H. Bai, W. Su, T. Li, Z. Zhang, R. Ma et al., Fullerene-embedded porphyrin metallacages as photoactive additives for stable binary organic solar cells with a certified efficiency of 20.2%. Energy Environ. Sci. **18**(24), 10351–10363 (2025). 10.1039/D5EE04824A

[18] D. Hu, H. Tang, J. Fu, Y. Li, L. Liu et al., Self-assembly control of Y-series non-fullerene acceptors for sustainable and scalable organic photovoltaics. Nano-Micro Lett. **18**(1), 182 (2026). 10.1007/s40820-025-02021-7 10.1007/s40820-025-02021-7 PMC12765764 41486244

[19] C. He, Z. Chen, T. Wang, Z. Shen, Y. Li et al., Asymmetric electron acceptor enables highly luminescent organic solar cells with certified efficiency over 18%. Nat. Commun. **13**, 2598 (2022). 10.1038/s41467-022-30225-7 35545620 10.1038/s41467-022-30225-7 PMC9095617

[20] C. Li, J. Zhou, J. Song, J. Xu, H. Zhang et al., Non-fullerene acceptors with branched side chains and improved molecular packing to exceed 18% efficiency in organic solar cells. Nat. Energy **6**(6), 605–613 (2021). 10.1038/s41560-021-00820-x

[21] D. Li, N. Wei, Y.-N. Chen, X. Wang, X. Han et al., Asymmetric side-group engineering of nonfused ring electron acceptors for high-efficiency thick-film organic solar cells. Nano-Micro Lett. **18**(1), 81 (2025). 10.1007/s40820-025-01905-y 10.1007/s40820-025-01905-y PMC12597859 41207984

[22] B. Zou, W. Wu, T.A. Dela Peña, R. Ma, Y. Luo et al., Step-by-step modulation of crystalline features and exciton kinetics for 19.2% efficiency ortho-xylene processed organic solar cells. Nano-Micro Lett. **16**(1), 30 (2023). 10.1007/s40820-023-01241-z 10.1007/s40820-023-01241-z PMC10667184 37995001

[23] L. Xue, Q. Xie, W. Xie, Y. Fu, P. Zhu et al., Precise side-chain engineering optimizes polymer pre-aggregation and crystallinity for efficient organic solar cells with minimized non-radiative energy loss. Aggregate **6**(9), e70103 (2025). 10.1002/agt2.70103

[24] J. Wang, P. Wang, T. Chen, W. Zhao, J. Wang et al., Isomerism effect of 3D dimeric acceptors for non-halogenated solvent-processed organic solar cells with 20 % efficiency. Angew. Chem. Int. Ed. **64**(12), e202423562 (2025). 10.1002/anie.202423562 10.1002/anie.202423562 39723552

[25] H. Fang, C. Xiao, S. Liang, L. Liu, J. Huang et al., Long-lived charge-transfer state and interfacial lock in double-cable conjugated polymers enable efficient and stable organic solar cells. Angew. Chem. **137**(45), e202514735 (2025). 10.1002/ange.202514735 10.1002/anie.202514735 40931586

[26] D. Hu, H. Tang, C. Chen, P. Huang, Z. Shen et al., Insights into preaggregation control of *Y*-series nonfullerene acceptors in liquid state for highly efficient binary organic solar cells. Adv. Mater. **36**(30), 2402833 (2024). 10.1002/adma.202402833 10.1002/adma.202402833 38837820

[27] H. Xia, Y. Zhang, K. Liu, W. Deng, M. Zhu et al., Oligomeric semiconductors enable high efficiency open air processed organic solar cells by modulating pre-aggregation and crystallization kinetics. Energy Environ. Sci. **16**(12), 6078–6093 (2023). 10.1039/D3EE02984K

[28] J. Zhao, S. Zhao, Z. Xu, B. Qiao, D. Huang et al., Revealing the effect of additives with different solubility on the morphology and the donor crystalline structures of organic solar cells. ACS Appl. Mater. Interfaces **8**(28), 18231–18237 (2016). 10.1021/acsami.6b02671 27328855 10.1021/acsami.6b02671

[29] S. Xiong, Y. Zhu, Y. Wang et al., Controlling morphology and improving stability with high-boiling-point additive for efficient organic solar cells. ACS Appl. Mater. Interfaces **17**(12), 18473–18481 (2025). 10.1021/acsami.5c01134 40094444 10.1021/acsami.5c01134

[30] Y. Zhou, W. Su, Z. Liang, Q. Wu et al., Halogenated diphenyl ether solvent additives enable ∼20% efficiency organic solar cells and high-performance opaque/semitransparent modules. Natl. Sci. Rev. **12**(10), nwaf346 (2025). 10.1093/nsr/nwaf346 41040499 10.1093/nsr/nwaf346 PMC12485983

[31] K. Sun, Y. Wang, G. Zhang, C. Gao, X. Ling et al., 20.64% efficient and stable binary organic solar cells *via* thermodynamic-engineered interlayer diffusion and exciton generation. Adv. Mater. **37**(47), e09806 (2025). 10.1002/adma.202509806 40884175 10.1002/adma.202509806

[32] D. Leman, M.A. Kelly, S. Ness, S. Engmann, A. Herzing et al., In situ characterization of polymer–fullerene bilayer stability. Macromolecules **48**(2), 383–392 (2015). 10.1021/ma5021227

[33] E.M. Pearce, Properties of polymers, their estimation and correlation with chemical structure–(2nd rev. Ed.), D. W. Van Krevelen, Elsevier, Amsterdam–Oxford–New York, 1976, 620 pp. J. Polym. Sci. B Polym. Lett. Ed. **15**(1), 56 (1977). 10.1002/pol.1977.130150109

[34] F. Neese, Software update: The ORCA program system: Version 5.0. Wires Comput. Mol. Sci. **12**(5), e1606 (2022). 10.1002/wcms.1606

[35] T. Lu, Q. Chen, Independent gradient model based on Hirshfeld partition: A new method for visual study of interactions in chemical systems. J. Comput. Chem. **43**(8), 539–555 (2022). 10.1002/jcc.26812 35108407 10.1002/jcc.26812

[36] P. Müller-Buschbaum, The active layer morphology of organic solar cells probed with grazing incidence scattering techniques. Adv. Mater. **26**(46), 7692–7709 (2014). 10.1002/adma.201304187 24677365 10.1002/adma.201304187

[37] D. Mo, H. Chen, J. Zhou, N. Tang, L. Han et al., Alkyl chain engineering of chlorinated acceptors for elevated solar conversion. J. Mater. Chem. A **8**(18), 8903–8912 (2020). 10.1039/c9ta12558b

[38] T.M. Eggenhuisen, Y. Galagan, A.F.K.V. Biezemans, T.M.W.L. Slaats, W.P. Voorthuijzen et al., High efficiency, fully inkjet printed organic solar cells with freedom of design. J. Mater. Chem. A **3**(14), 7255–7262 (2015). 10.1039/c5ta00540j

[39] D. Corzo, K. Almasabi, E. Bihar, S. MacPhee, D. Rosas-Villalva et al., Digital inkjet printing of high-efficiency large-area nonfullerene organic solar cells. Adv. Mater. Technol. **4**(7), 1900040 (2019). 10.1002/admt.201900040

[40] S. Jung, A. Sou, K. Banger, D.-H. Ko, P.C.Y. Chow et al., All-inkjet-printed, all-air-processed solar cells. Adv. Energy Mater. **4**(14), 1400432 (2014). 10.1002/aenm.201400432

[41] C.N. Hoth, S.A. Choulis, P. Schilinsky, C.J. Brabec, High photovoltaic performance of inkjet printed polymer: fullerene blends. Adv. Mater. **19**(22), 3973–3978 (2007). 10.1002/adma.200700911

[42] D. Corzo, E. Bihar, E.B. Alexandre, D. Rosas-Villalva, D. Baran, Ink engineering of transport layers for 9.5% efficient all-printed semitransparent nonfullerene solar cells. Adv. Funct. Mater. **31**(7), 2005763 (2021). 10.1002/adfm.202005763

[43] X. Chen, R. Huang, Y. Han, W. Zha, J. Fang et al., Balancing the molecular aggregation and vertical phase separation in the polymer: nonfullerene blend films enables 13.09% efficiency of organic solar cells with inkjet-printed active layer. Adv. Energy Mater. **12**(12), 2200044 (2022). 10.1002/aenm.202200044

[44] C.N. Hoth, S.A. Choulis, P. Schilinsky, C.J. Brabec, On the effect of poly(3-hexylthiophene) regioregularity on inkjet printed organic solar cells. J. Mater. Chem. **19**(30), 5398–5404 (2009). 10.1039/B823495G

[45] A. Lange, M. Wegener, C. Boeffel, B. Fischer, A. Wedel et al., A new approach to the solvent system for inkjet-printed P3HT: PCBM solar cells and its use in devices with printed passive and active layers. Sol. Energy Mater. Sol. Cells **94**(10), 1816–1821 (2010). 10.1016/j.solmat.2010.05.054

[46] H. Liu, L. Tang, T. Li, F. Yi, W. Su et al., Hybrid linking sites constructed non-fully conjugated asymmetric dimerized giant molecule acceptors for organic solar cells with an efficiency of ≈20%. Angew. Chem. Int. Ed. **64**(19), e202503721 (2025). 10.1002/anie.202503721 10.1002/anie.202503721 40029156

[47] T. Li, W. Zhou, H. Li, T. Wang, W. Su et al., Trimeric acceptors with fine-tuned alkyl-linkage sites for 20.23% efficiency and stable organic solar cells. Angew. Chem. Int. Ed. **65**(12), e6120368 (2026). 10.1002/anie.6120368 10.1002/anie.6120368 41645862

[48] X. Jiang, A.J. Gillett, T. Zheng, X. Song, J.E. Heger et al., Operando study of the influence of small molecule acceptors on the morphology induced device degradation of organic solar cells with different degrees of π–π stacking. Energy Environ. Sci. **16**(12), 5970–5981 (2023). 10.1039/D3EE02527F

[49] J. Song, C. Zhang, C. Li, J. Qiao, J. Yu et al., Non-halogenated solvent-processed organic solar cells with approaching 20 % efficiency and improved photostability. Angew. Chem. Int. Ed. **63**(22), e202404297 (2024). 10.1002/anie.202404297 10.1002/anie.202404297 38526996

[50] Z. Zhao, S. Chung, Y.Y. Kim, M. Jeong, X. Li et al., Room-temperature-modulated polymorphism of nonfullerene acceptors enables efficient bilayer organic solar cells. Energy Environ. Sci. **17**(15), 5666–5678 (2024). 10.1039/D4EE02330G

[51] Z. Bi, C. Liu, W. Ma, In situ morphology control for solution-printable organic photovoltaics. Adv. Funct. Mater. **34**(49), 2409315 (2024). 10.1002/adfm.202409315

[52] H. Zhang, C. Tian, Z. Zhang, M. Xie, J. Zhang et al., Concretized structural evolution supported assembly-controlled film-forming kinetics in slot-die coated organic photovoltaics. Nat. Commun. **14**, 6312 (2023). 10.1038/s41467-023-42018-7 37813858 10.1038/s41467-023-42018-7 PMC10562442

[53] Y. Fu, L. Wang, C. Guo, D. Li, J. Cai et al., Side chain length and interaction mediated charge transport networks of non-fullerene acceptors for efficient organic solar cells. ACS Materials Letters **4**(10), 2009–2018 (2022). 10.1021/acsmaterialslett.2c00764

[54] X. Zhang, H. Wang, D. Li, M. Chen, Y. Mao et al., Modulation of J-aggregation of nonfullerene acceptors toward near-infrared absorption and enhanced efficiency. Macromolecules **53**(10), 3747–3755 (2020). 10.1021/acs.macromol.0c00469

[55] T. Lu, F. Chen, Multiwfn: a multifunctional wavefunction analyzer. J. Comput. Chem. **33**(5), 580–592 (2012). 10.1002/jcc.22885 22162017 10.1002/jcc.22885

[56] T. Lu, S. Manzetti, Wavefunction and reactivity study of benzo [a] pyrene diol epoxide and its enantiomeric forms. Struct. Chem. **25**(5), 1521–1533 (2014). 10.1007/s11224-014-0430-6

[57] J. Yao, T. Kirchartz, M.S. Vezie, M.A. Faist, W. Gong et al., Quantifying losses in open-circuit voltage in solution-processable solar cells. Phys. Rev. Appl. **4**, 014020 (2015). 10.1103/physrevapplied.4.014020

[58] K. Vandewal, S. Albrecht, E.T. Hoke, K.R. Graham, J. Widmer et al., Efficient charge generation by relaxed charge-transfer states at organic interfaces. Nat. Mater. **13**(1), 63–68 (2014). 10.1038/nmat3807 24240240 10.1038/nmat3807

[59] F. Gao, O. Inganäs, Charge generation in polymer-fullerene bulk-heterojunction solar cells. Phys. Chem. Chem. Phys. **16**(38), 20291–20304 (2014). 10.1039/c4cp01814a 24994122 10.1039/c4cp01814a

[60] J. Yuan, C. Zhang, H. Chen, C. Zhu, S.H. Cheung et al., Understanding energetic disorder in electron-deficient-core-based non-fullerene solar cells. Sci. China Chem. **63**(8), 1159–1168 (2020). 10.1007/s11426-020-9747-9

[61] S. Xie, Y. Xia, Z. Zheng, X. Zhang, J. Yuan et al., Effects of nonradiative losses at charge transfer states and energetic disorder on the open-circuit voltage in nonfullerene organic solar cells. Adv. Funct. Mater. **28**(5), 1705659 (2018). 10.1002/adfm.201705659

[62] Y. Qin, S. Zhang, Y. Xu, L. Ye, Y. Wu et al., Reduced nonradiative energy loss caused by aggregation of nonfullerene acceptor in organic solar cells. Adv. Energy Mater. **9**(35), 1901823 (2019). 10.1002/aenm.201901823

[63] X.-K. Chen, M.K. Ravva, H. Li, S.M. Ryno, J.-L. Brédas, Effect of molecular packing and charge delocalization on the nonradiative recombination of charge-transfer states in organic solar cells. Adv. Energy Mater. **6**(24), 1601325 (2016). 10.1002/aenm.201601325

[64] N. An, Y. Cai, H. Wu, A. Tang, K. Zhang et al., Solution-processed organic solar cells with high open-circuit voltage of 1.3 V and low non-radiative voltage loss of 0.16 V. Adv. Mater. **32**(39), 2002122 (2020). 10.1002/adma.202002122 10.1002/adma.202002122 32844465

[65] Y. Shi, L. Zhu, Y. Yan, M. Xie, G. Liang et al., Small energetic disorder enables ultralow energy losses in non-fullerene organic solar cells. Adv. Energy Mater. **13**(19), 2300458 (2023). 10.1002/aenm.202300458

[66] F. Urbach, The long-wavelength edge of photographic sensitivity and of the electronic absorption of solids. Phys. Rev. **92**(5), 1324 (1953). 10.1103/physrev.92.1324

[67] J. Wu, J. Luke, H.K.H. Lee, P. Shakya Tuladhar, H. Cha et al., Tail state limited photocurrent collection of thick photoactive layers in organic solar cells. Nat. Commun. **10**, 5159 (2019). 10.1038/s41467-019-12951-7 31727897 10.1038/s41467-019-12951-7 PMC6856365

[68] G. Garcia-Belmonte, P.P. Boix, J. Bisquert, M. Lenes, H.J. Bolink et al., Influence of the intermediate density-of-states occupancy on open-circuit voltage of bulk heterojunction solar cells with different fullerene acceptors. J. Phys. Chem. Lett. **1**(17), 2566–2571 (2010). 10.1021/jz100956d

[69] Z. Zhang, Y. Li, G. Cai, Y. Zhang, X. Lu et al., Selenium heterocyclic electron acceptor with small urbach energy for As-cast high-performance organic solar cells. J. Am. Chem. Soc. **142**(44), 18741–18745 (2020). 10.1021/jacs.0c08557 33085460 10.1021/jacs.0c08557

[70] J. Yuan, C. Zhang, B. Qiu, W. Liu, S.K. So et al., Effects of energetic disorder in bulk heterojunction organic solar cells. Energy Environ. Sci. **15**(7), 2806–2818 (2022). 10.1039/d2ee00271j

[71] J. Yuan, Y. Zhang, L. Zhou, C. Zhang, T.-K. Lau et al., Fused benzothiadiazole: a building block for n-type organic acceptor to achieve high-performance organic solar cells. Adv. Mater. **31**(17), 1807577 (2019). 10.1002/adma.201807577 10.1002/adma.201807577 30883937

[72] D.T. Duong, B. Walker, J. Lin, C. Kim, J. Love et al., Molecular solubility and Hansen solubility parameters for the analysis of phase separation in bulk heterojunctions. J. Polym. Sci. Part B Polym. Phys. **50**(20), 1405–1413 (2012). 10.1002/polb.23153

