# Supplementary material for: Droplet-Merging and Dissolution-Induced Intermediate State Strategy Enabled Efficiency > 17.5% for the Printed Organic Solar Cells
Source: Nanomicro Lett. 2026 Jul 6;18:425. doi: 10.1007/s40820-026-02268-8 (PMC13337997; doi:10.1007/s40820-026-02268-8)
Supplement: Supplementary file 1 — Supplementary file1 (DOCX 6534 kb) [file 40820_2026_2268_MOESM1_ESM.docx]

Supporting Information for

**Droplet Merging and Dissolution-Induced** **Intermediate State Strategy Enabled Efficiency > 17.5% for the Printed Organic Solar Cells**

Lifeng Sang^1, 2^, Xingze Chen^2^, Chen Chen^3^, Yuanyuan Jiang^4^, Qing Zhang^5^, Ni Yin^2^, Yue Guo^5^, Wei Li^3,^ *, Tao Wang^3^, Xiaozhang Zhu^4,^ *, Qi Chen^1, 2,^ *, Chang-Qi Ma^1, 2,^ *, Qun Luo^1, 2,^ *

^1^ School of Nano-Tech and Nano-Bionics, University of Science and Technology of China, Hefei 230027, P. R. China

^2^ i-Lab, Suzhou Institute of Nano-Tech and Nano-Bionics, Chinese Academy of Sciences (CAS), Suzhou 215123, P. R. China

^3^ School of Materials Science and Engineering, Wuhan University of Technology, Wuhan, 430070, P. R. China

^4^ Beijing National Laboratory for Molecular Sciences, CAS Key Laboratory of Organic Solids, Chinese Academy of Sciences (CAS), Beijing 100190, P. R. China

^5^ Vacuum Interconnected Nanotech Workstation (Nano-X), Suzhou Institute of Nano-Tech and Nano-Bionics, Chinese Academy of Sciences (CAS), Suzhou 215123, P. R. China

*Corresponding authors. E-mail: [liwei1992@whut.edu.cn](mailto:liwei1992@whut.edu.cn) (Wei Li); [xzzhu@iccas.ac.cn](mailto:xzzhu@iccas.ac.cn) (Xiaozhang Zhu); [qchen2011@sinano.ac.cn](mailto:qchen2011@sinano.ac.cn) (Qi Chen); [cqma2011@sinano.ac.cn](mailto:cqma2011@sinano.ac.cn) (Chang-Qi Ma); [qluo2011@sinano.ac.cn](mailto:qluo2011@sinano.ac.cn) (Qun Luo)

**Supplementary Figures and Tables**


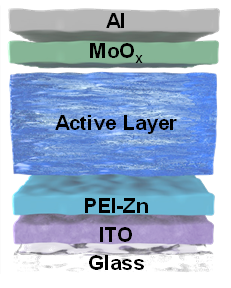


**Fig. S1** The schematic diagram of the device architecture.

**Table S1** Photovoltaic parameters of spin-coated OSCs, fabricated though different solvents, under AM1.5G 100 mW/cm^2^ illumination

| Condition | *V*_OC_ (V) | *J*_SC_ (mA/cm^2^) | FF (%) | PCE (%) |
| --- | --- | --- | --- | --- |
| CF | 0.832 | 26.57 | 73.79 | 16.31 |
|  | 0.829±0.003 | 25.95±0.62 | 73.84±0.59 | 15.88±0.40 |
| oDCB | 0.832 | 24.24 | 71.58 | 14.43 |
|  | 0.835±0.006 | 24.08±0.11 | 70.97±0.53 | 14.27±0.12 |


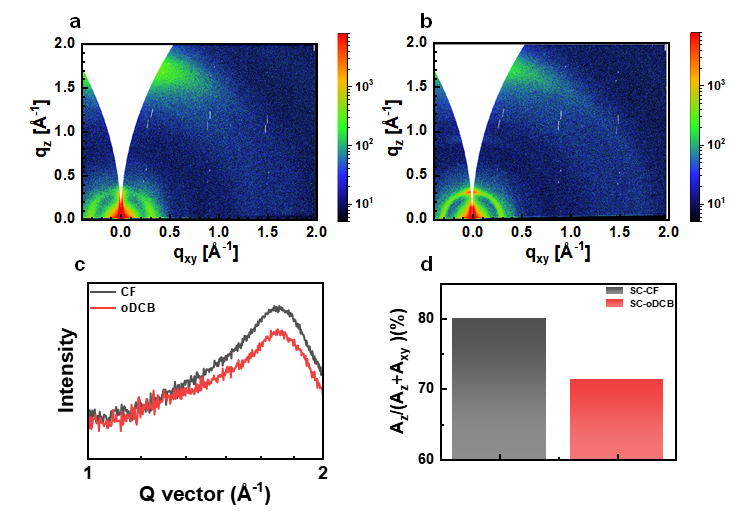


**Fig. S2** 2D-GIWAXS patterns of the spin-coated films processed from **a** CF, and **b** oDCB. **c** Corresponding line-cut profiles in the OOP directions of the 010 peak. **d** The face-on ratio calculated from the pole figure of the 010 peak.

**
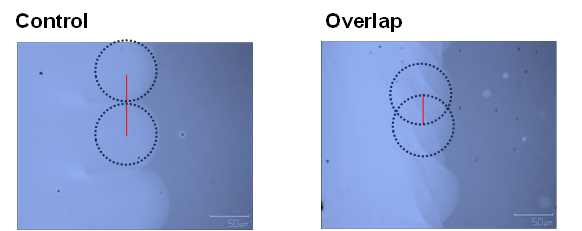
**

**Fig. S3** Microscope images of films prepared by printing under different conditions.


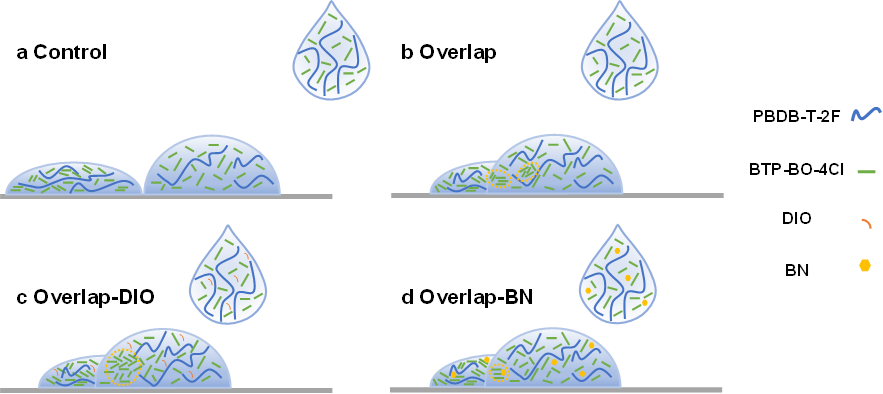


**Fig. S4** The schematic diagram of the films prepared by printing under different conditions. **a** Control, **b** Overlap, **c** Overlap-DIO, **d** Overlap-BN, respectively.


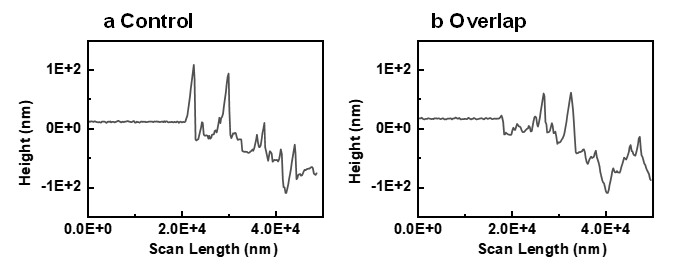


**Fig. S5** Thickness test for films prepared by printing under different conditions. **a** Control, and **b** Overlap.


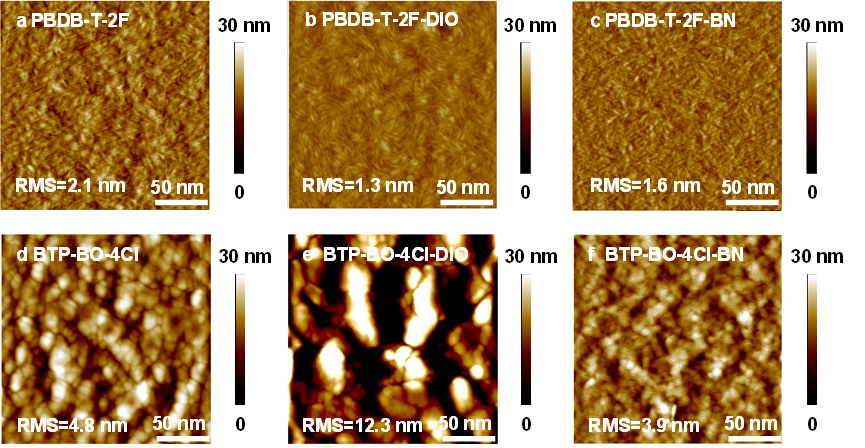


**Fig. S6** AFM height images for printed (**a**-**c**) PBDB-T-2F neat films and (**d**-**f**) BTP-BO-4Cl neat films fabricated though different additives.


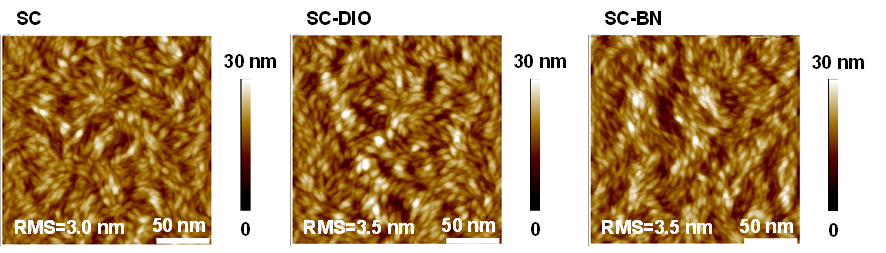


**Fig. S7** The AFM height images of blend PBDB-T-2F:BTP-BO-4Cl films fabricated by spin-coating.

**
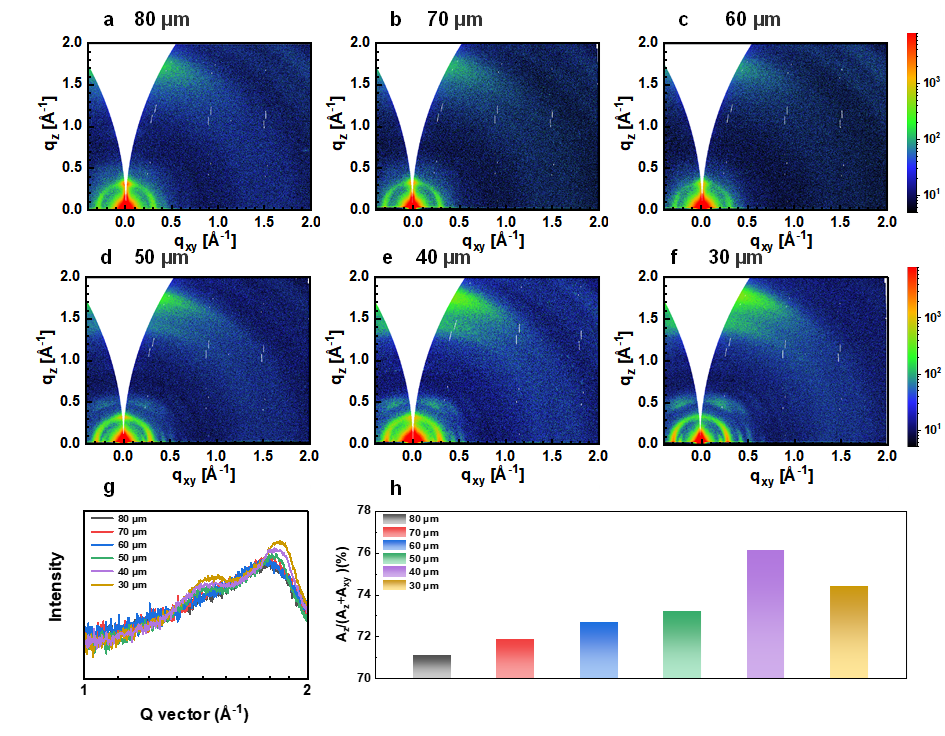
**

**Fig. S8** 2D-GIWAXS patterns of the printed films processed from **a** 80 µm, **b** 70 µm, **c** 60 µm, **d** 50 µm, **e** 40 µm, and **f** 30 µm. **g** Corresponding line-cut profiles in the OOP directions of the 010 peak. **h** The face-on ratio calculated from the pole figure of the 010 peak.


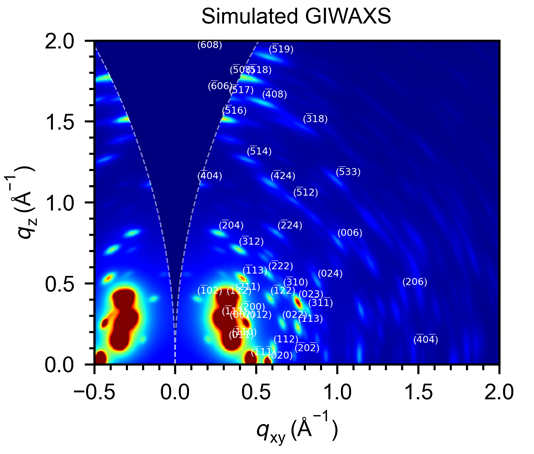


**Fig. S9** Simulated GIWAXS.


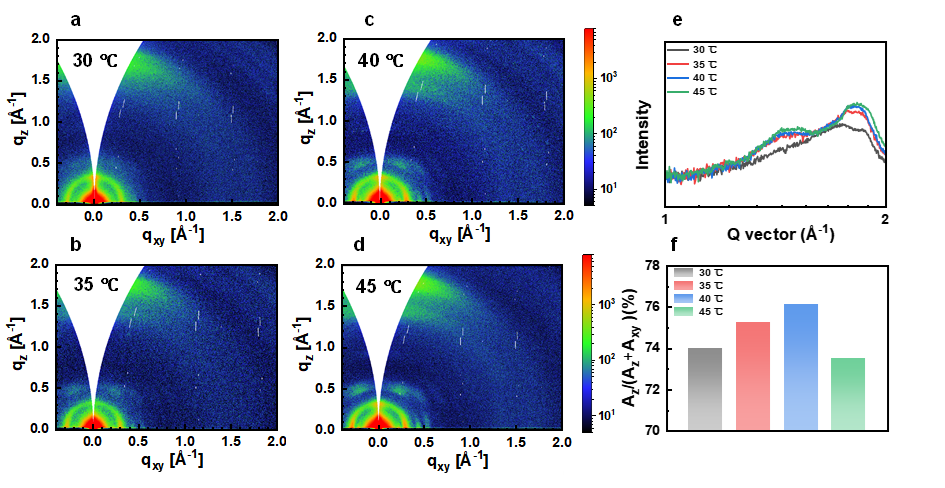


**Fig. S10** 2D-GIWAXS patterns of the printed films processed from **a** 30 ℃, **b** 35 ℃, **c** 40 ℃, and **d** 45 ℃. **e** Corresponding line-cut profiles in the OOP directions of the 010 peak. **f** The face-on ratio calculated from the pole figure of the 010 peak.


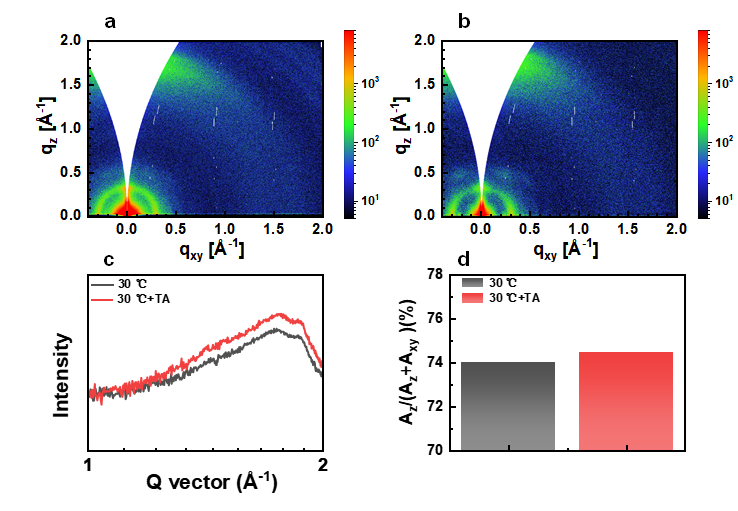


**Fig. S11** 2D-GIWAXS patterns of the printed films processed *via* printing on **a** a substrate at 30 ℃, and **b** a substrate at 30 ℃ followed by thermal annealing (30 ℃+TA). **c** Corresponding line-cut profiles in the OOP directions of the 010 peak. **d** The face-on ratio calculated from the pole figure of the 010 peak.

**Table S2** GIWAXS parameters in the in-plane direction of printed films fabricated using different processing methods, including q, d-spacing, FWHM, and CCLs

|  |  | Location(Å^-1^) | d-spacing(Å) | FWHM(Å^-1^) | CCL(Å) |
| --- | --- | --- | --- | --- | --- |
| IP | Control |  |  |  |  |
|  | Overlap | 0.21 | 29.40 | 0.04 | 149.60 |
|  | Overlap-DIO | 0.22 | 28.81 | 0.04 | 141.94 |
|  | Overlap-BN |  |  |  |  |
|  | Control | 0.29 | 21.32 | 0.06 | 101.76 |
|  | Overlap | 0.29 | 21.92 | 0.04 | 128.66 |
|  | Overlap-DIO | 0.29 | 21.91 | 0.04 | 144.22 |
|  | Overlap-BN | 0.30 | 21.10 | 0.05 | 118.59 |
|  | Control |  |  |  |  |
|  | Overlap | 0.42 | 14.85 | 0.06 | 94.09 |
|  | Overlap-DIO | 0.43 | 14.77 | 0.04 | 156.65 |
|  | Overlap-BN |  |  |  |  |
|  | Control |  |  |  |  |
|  | Overlap | 0.52 | 12.17 | 0.06 | 101.04 |
|  | Overlap-DIO | 0.52 | 12.13 | 0.06 | 92.76 |
|  | Overlap-BN |  |  |  |  |

**Table S3** GIWAXS parameters in the out-of-plane direction of printed films fabricated using different processing methods, including q, d-spacing, FWHM, and CCLs

|  |  | Location(Å^-1^) | d-spacing(Å) | FWHM(Å^-1^) | CCL(Å) |
| --- | --- | --- | --- | --- | --- |
| OOP | Control | 0.32 | 19.68 | 0.07 | 84.52 |
|  | Overlap | 0.32 | 19.85 | 0.08 | 69.61 |
|  | Overlap-DIO | 0.31 | 20.26 | 0.05 | 118.12 |
|  | Overlap-BN | 0.32 | 19.83 | 0.07 | 86.51 |
|  | Control |  |  |  |  |
|  | Overlap | 0.52 | 12.08 | 0.11 | 51.76 |
|  | Overlap-DIO | 0.52 | 12.09 | 0.05 | 118.12 |
|  | Overlap-BN |  |  |  |  |
|  | Control | 0.96 | 6.57 | 0.59 | 9.53 |
|  | Overlap |  |  |  |  |
|  | Overlap-DIO |  |  |  |  |
|  | Overlap-BN | 0.94 | 6.67 | 0.06 | 98.88 |
|  | Control |  |  |  |  |
|  | Overlap | 1.50 | 4.17 | 0.35 | 16.09 |
|  | Overlap-DIO | 1.49 | 4.21 | 0.40 | 14.30 |
|  | Overlap-BN |  |  |  |  |
|  | Control | 1.76 | 3.56 | 0.45 | 12.45 |
|  | Overlap | 1.81 | 3.51 | 0.21 | 26.29 |
|  | Overlap-DIO | 1.83 | 3.48 | 0.83 | 6.84 |
|  | Overlap-BN | 1.77 | 3.52 | 0.17 | 33.56 |

**
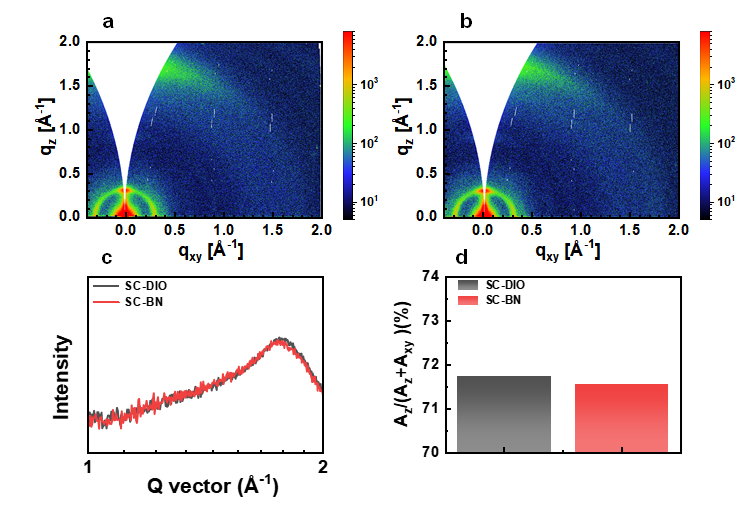
**

**Fig. S12** 2D-GIWAXS patterns of the spin-coated films processed with **a** DIO (SC-DIO), and **b** BN (SC-BN). **c** Corresponding line-cut profiles in the OOP directions of the 010 peak. **d** The face-on ratio calculated from the pole figure of the 010 peak.


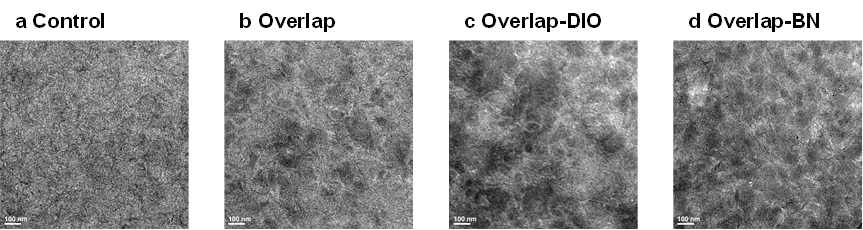


**Fig. S13** TEM images of the printed films processed under **a** Control, **b** Overlap, **c** Overlap-DIO, and **d** Overlap-BN, respectively.


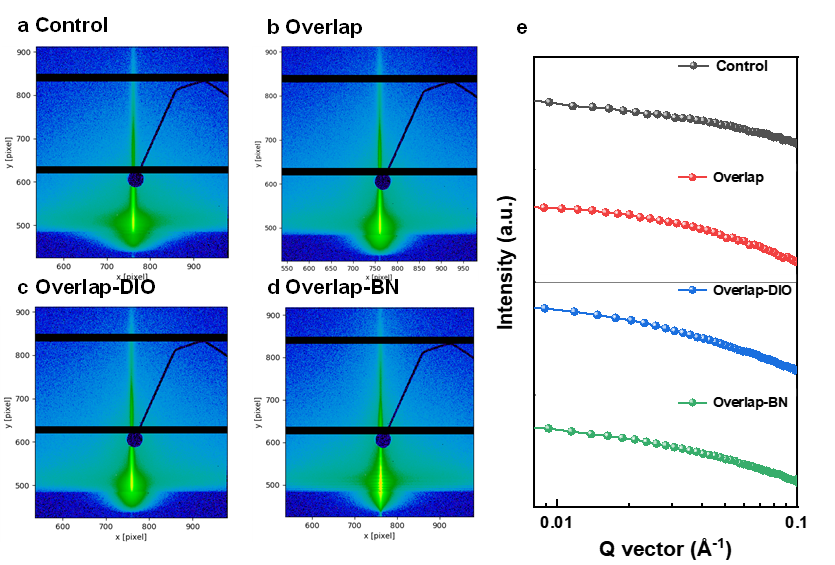


**Fig. S14** 2D GISAXS patterns of the printed films processed under **a** Control, **b** Overlap, **c** Overlap-DIO, and **d** Overlap-BN, respectively. **e** GISAXS intensity profiles of the corresponding films along the Q axis.

**Table S4**. Morphology parameters fitted by the GISAXS profiles

| Sample | Large domain size  (nm) | Middle domain size  (nm) | Small domain size  (nm) |
| --- | --- | --- | --- |
| Control | 25.9±0.7 | 18.9±0.6 | 7.9±0.7 |
| Overlap | 58.4±1.0 | 23.5±0.8 | 18.7±0.7 |
| Overlap-DIO | 88.5±0.9 | 35.8±0.6 | 31.1±1.1 |
| Overlap-BN | 40.4±0.7 | 20.3±1.0 | 9.8±0.4 |


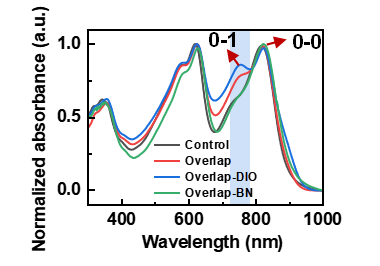


**Fig. S15** Normalized UV-vis absorption spectra of printed films fabricated though different processing methods.


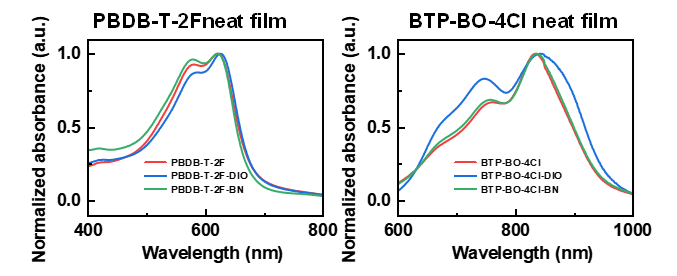


**Fig. S16** Normalized UV-vis absorption spectra of printed PBDB-T-2F neat films and BTP-BO-4Cl neat films fabricated though different additives.


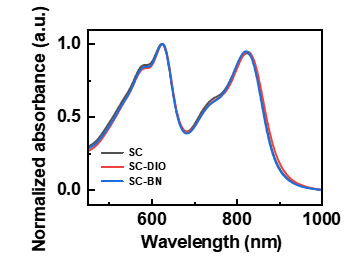


**Fig. S17** Normalized UV-vis absorption spectra of spin-coated films fabricated though different additives.


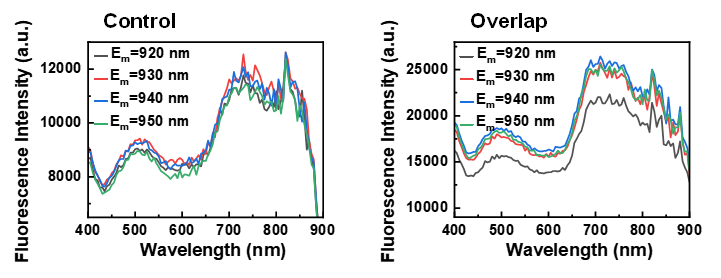


**Fig. S18** PLE spectroscopy patterns of the blend films processed from Control, and Overlap.


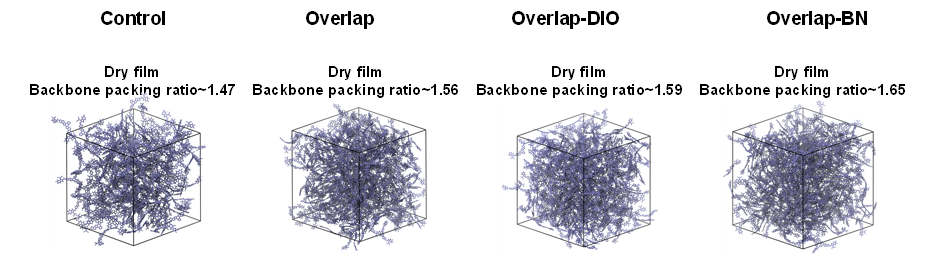


**Fig. S19** Results of molecular dynamics simulations (MDS) processed from Control, Overlap, Overlap-DIO, and Overlap-BN, respectively.

**Table S5** The resulting molecular packing parameters.

| Condition | Backbone  atoms | Backbone  packing units | Backbone  packing ratio | Alkyl chain  atoms | Alkyl chain  packing units | Alkyl chain  packing ratio |
| --- | --- | --- | --- | --- | --- | --- |
| Control |  |  | 1.47 |  |  | 1.84 |
| Redissolved |  |  | 1.56 |  |  | 1.91 |
| Redissolved with DIO | 27600 | 43766 | 1.59 | 18400 | 39748 | 2.16 |
| Redissolved with BN | 27600 | 45396 | 1.65 | 18400 | 36496 | 1.98 |


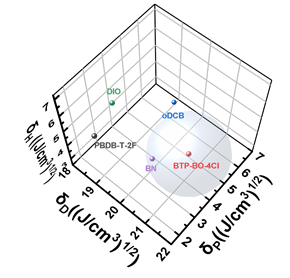


**Fig. S20** Calculated Flory-Huggins interaction parameter.

**Table S6** Calculated Flory-Huggins interaction parameter

| Condition | δ_D_ | δ_P_ | δ_H_ | Molar Volume  (cm^3^ /mol) | χ_PBDB-T-2F-Solvenr_ | χ_BTP-BO-4Cl-Solvenr_ | Boiling point  (℃) |
| --- | --- | --- | --- | --- | --- | --- | --- |
| PBDB-T-2F | 18.8 | 2.1 | 5.1 | 985.5 | / | / | / |
| BTP-BO-4Cl | 21.7 | 2.8 | 7.2 | 1252.0 | / | / | / |
| ortho-dichlorobenzene | 19.2 | 6.3 | 3.3 | 112.8 | 0.25 | 0.60 | 182 |
| 1,8-Diiodooctane | 18.4 | 3.7 | 5.0 | 192.0 | 0.06 | 0.95 | 333 |
| 1-Bromonaphthalene | 20.3 | 3.1 | 4.1 | 140.0 | 0.16 | 0.25 | 281 |

**Table S7** Performance parameters of inverted PBDB-T-2F:BTP-BO-4Cl OSCs with active layers fabricated from inkjet printing at different deposition temperatures

| Temperatures  (℃) | *V*_OC_  (V) | *J*_SC_  (mA/cm^2^) | FF  (%) | PCE  (%) |
| --- | --- | --- | --- | --- |
| 30 | 0.807 | 23.68 | 54.70 | 10.45 |
|  | 0.801±0.005 | 22.41±0.92 | 54.34±2.29 | 9.77±0.72 |
| 35 | 0.806 | 23.83 | 65.07 | 12.49 |
|  | 0.809±0.005 | 23.11±0.98 | 62.82±1.23 | 12.08±0.36 |
| 40 | 0.814 | 23.80 | 68.83 | 13.34 |
|  | 0.814±0.002 | 23.32±0.43 | 69.14±1.05 | 13.14±0.18 |
| 45 | 0.814 | 22.33 | 67.75 | 12.32 |
|  | 0.811±0.004 | 21.43±0.66 | 68.17±0.95 | 11.85±0.46 |


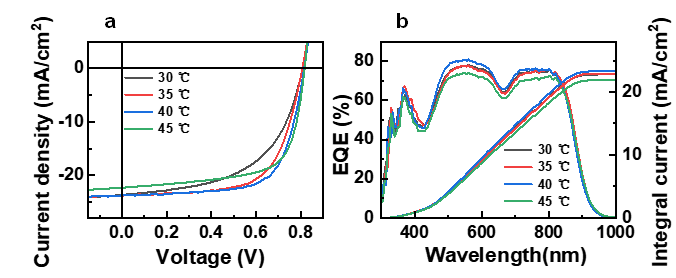


**Fig. S21 a** *J-V* characteristics, **b** EQE spectra of the inkjet-printed devices from different temperatures.


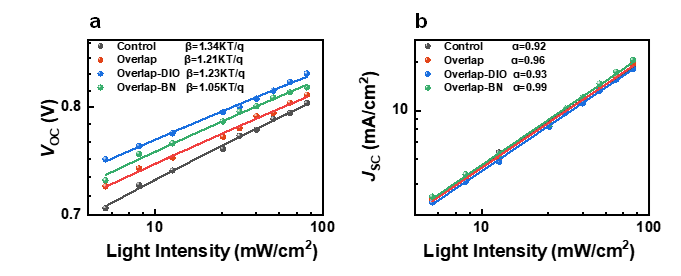


**Fig. S22 a** *V*_OC_ as a function of light intensity, and **b** *J*_SC_ as a function of light intensity of PBDB-T-2F:BTP-BO-4Cl fabricated though different processing methods.


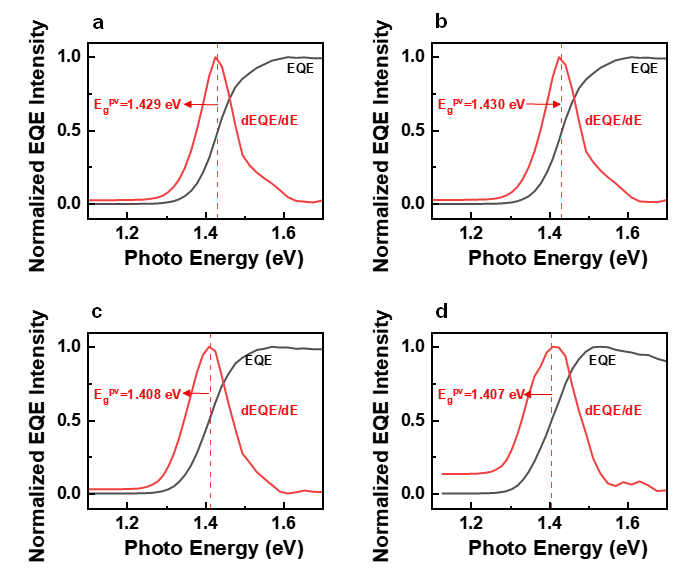


**Fig. S23** Calculation of the optical bandgap of the inkjet-printed films processed from **a** Control, **b** Overlap, **c** Overlap-DIO, and **d** Overlap-BN by using the maximum point of the derivative of the EQE spectra (dEQE/dE), respectively.


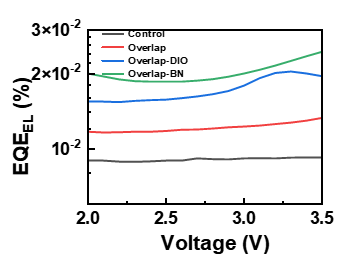


**Fig. S24** *EQE*_EL_ of PBDB-T-2F:BTP-BO-4Cl fabricated though different processing methods.


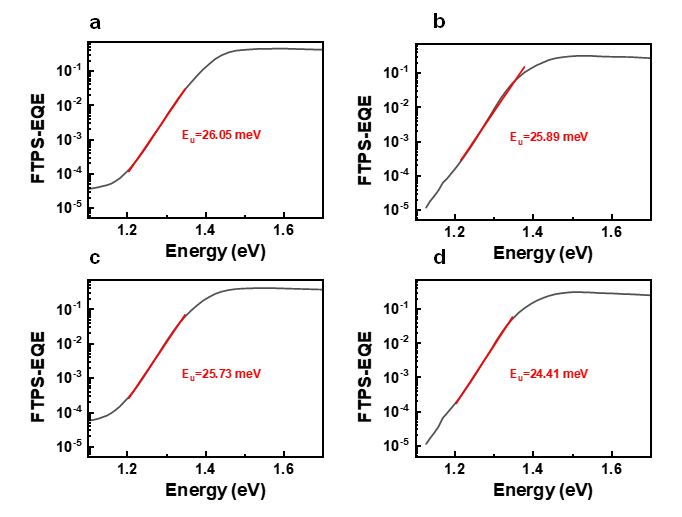


**Fig. S25** FTPS-EQE spectra of the corresponding inkjet-printed OSCs. **a** Control, **b** Overlap, **c** Overlap-DIO, and **d** Overlap-BN, respectively. *E*_u_ is Urbach energy, which is obtained from the FTPS-EQE curves with the exponential fitting(𝛼(𝐸) = 𝛼_0_ ∙ exp ((𝐸-𝐸_g_) /𝐸_u_)). where 𝛼(E) is the absorption coefficient, E is the photon energy, and 𝛼_0_ and *E*_g_ are two constants[S1, S2].


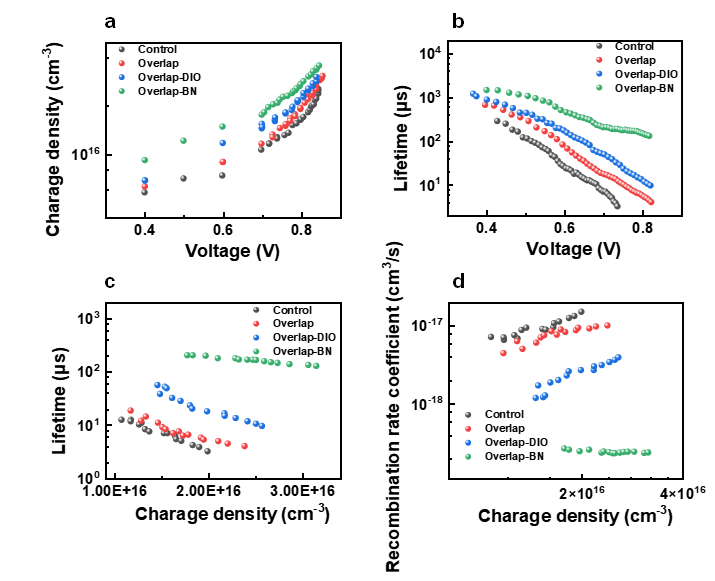


**Fig. S26 a** Lifetime and **b** charge-carriers density under different *V*_OC_ conditions. **c** Carrier lifetime versus charge density. **d** Bimolecular charge recombination rates (*k*_rec_) versus charge density of inkjet-printed OSCs fabricated though different processing methods.


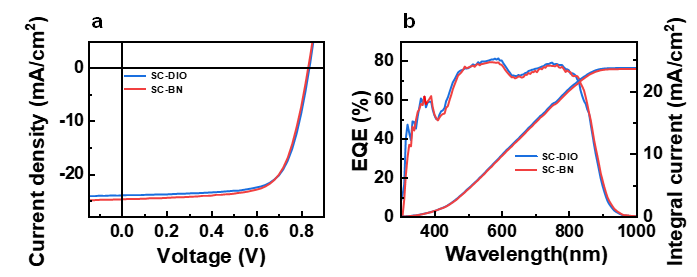


**Fig. S27** **a** *J-V* curves, **b** corresponding EQE spectra of the spin-coated devices though different additives.

**Table S8** Performance parameters of inverted OSCs with active layers fabricated from spin coating with different additive, under AM 1.5 G 100 mW/cm^2^ illumination

| Condition | *V*_OC_  (V) | *J*_SC_  (mA/cm^2^) | FF  (%) | PCE  (%) |
| --- | --- | --- | --- | --- |
| SC | 0.832 | 24.24 | 71.58 | 14.43 |
|  | 0.835±0.006 | 24.08±0.11 | 70.97±0.53 | 14.27±0.12 |
| SC-DIO | 0.836 | 24.03 | 71.30 | 14.33 |
|  | 0.824±0.06 | 24.23±0.27 | 70.21±1.33 | 14.03±0.28 |
| SC-BN | 0.831 | 24.79 | 70.07 | 14.44 |
|  | 0.833±0.04 | 24.52±0.27 | 68.94±1.52 | 14.08±0.41 |


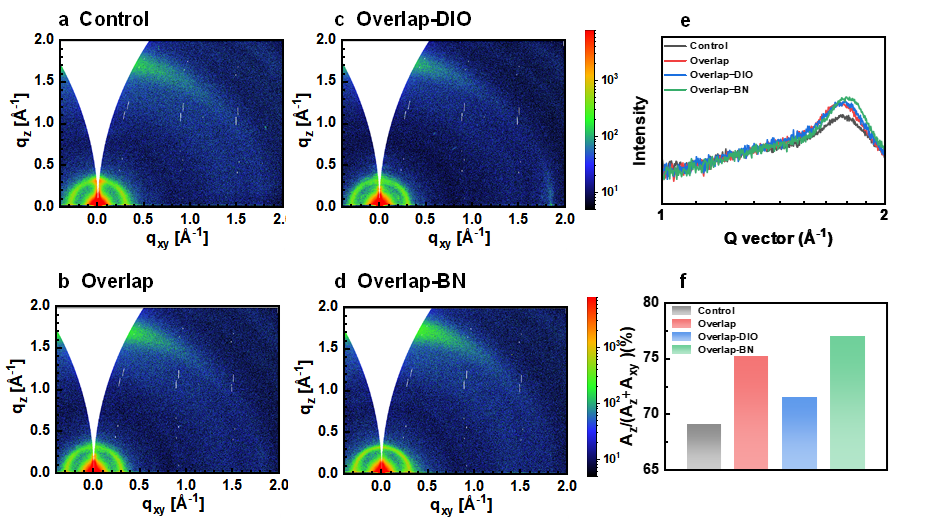


**Fig. S28** 2D-GIWAXS patterns of the PBDB-T-2F:N3 films processed from **a** Control, **b** Overlap, **c** Overlap-DIO, and **d** Overlap-BN, respectively. **e** Corresponding line-cut profiles in the OOP directions of the 010 peak. **f** The face-on ratio calculated from the pole figure of the 010 peak.


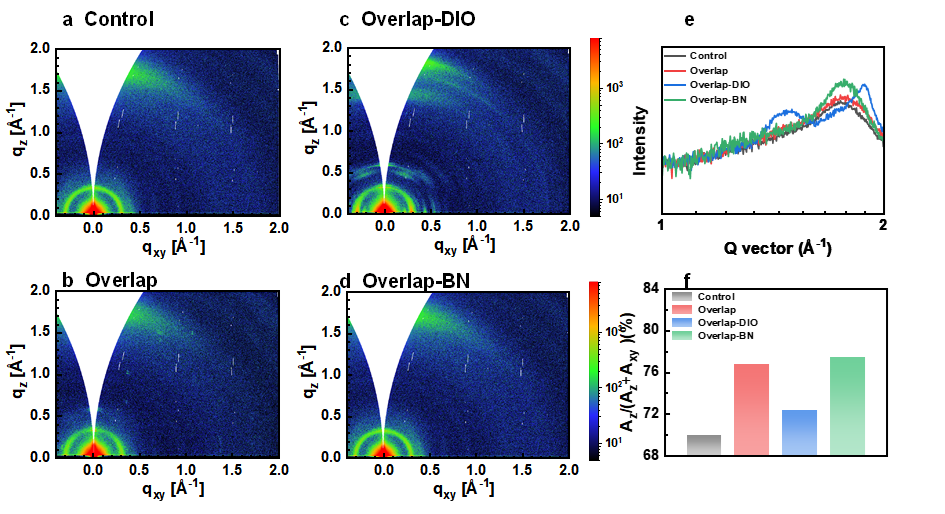


**Fig. S29** 2D-GIWAXS patterns of the PBDB-T-2F:BTP-eC9 films processed from **a** Control, **b** Overlap, **c** Overlap-DIO, and **d** Overlap-BN, respectively. **e** Corresponding line-cut profiles in the OOP directions of the 010 peak. **f** The face-on ratio calculated from the pole figure of the 010 peak.


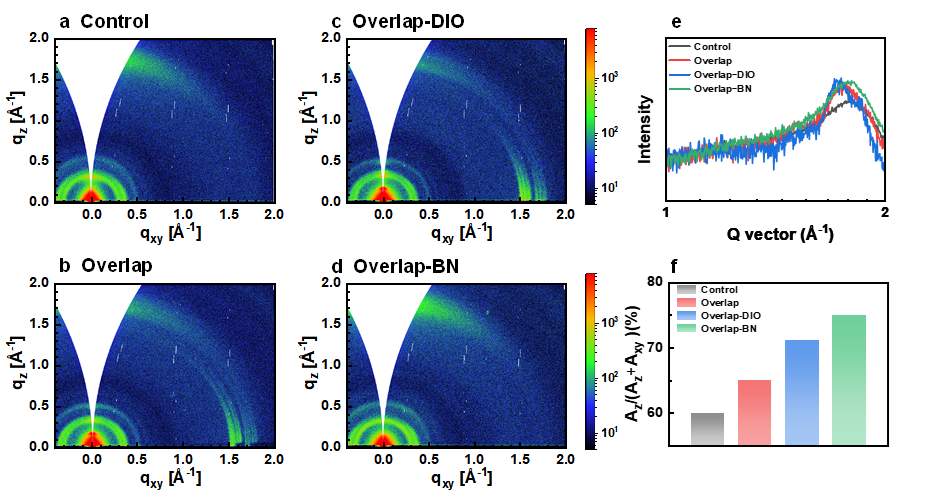


**Fig. S30**  2D-GIWAXS patterns of the PBDB-T-2F:L8-BO films processed from **a** Control, **b** Overlap, **c** Overlap-DIO, and **d** Overlap-BN, respectively. **e** Corresponding line-cut profiles in the OOP directions of the 010 peak. **f** The face-on ratio calculated from the pole figure of the 010 peak.


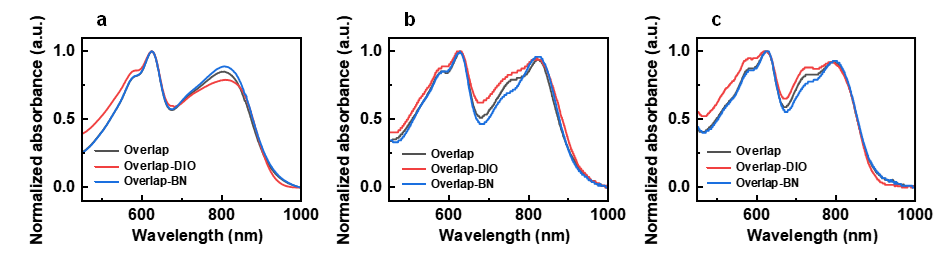


**Fig. S31** Normalized UV-vis absorption spectra of the films processed from **a** PBDB-T-2F:N3, **b** PBDB-T-2F:BTP-eC9, **c** PBDB-T-2F:L8-BO.


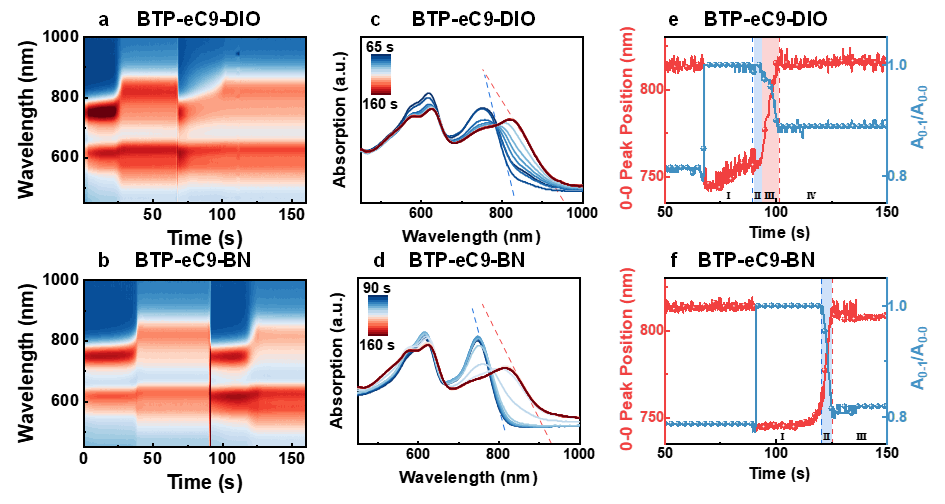


**Fig. S32**  In *situ* UV-vis absorption spectroscopy of the PBDB-T-2F:BTP-eC9 films processed with **a** DIO, and **b** BN. In *situ* UV-vis absorption of PBDB-T-2F:BTP-eC9films processed with **c** DIO, and **d** BN evolution from the solution to the film. Changes in the peak position and the 0-1/0-0 intensity ratio of BTP-eC9 processed with **e** DIO, and **f** BN.


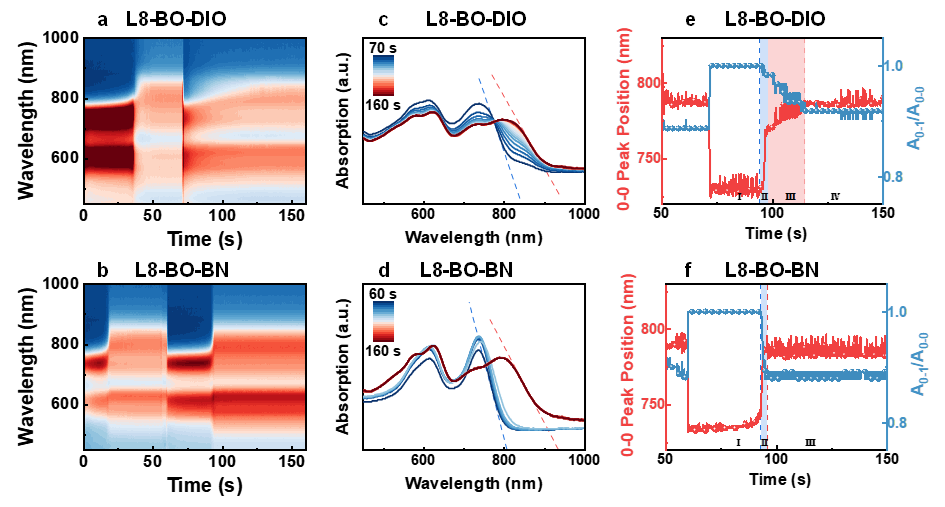


**Fig. S33** *In situ* UV-vis absorption spectroscopy of the PBDB-T-2F:L8-BO films processed with **a** DIO, and **b** BN. In *situ* UV-vis absorption of PBDB-T-2F:L8-BO films processed with **c** DIO, and **d** BN evolution from the solution to the film. Changes in the peak position and the 0-1/0-0 intensity ratio of L8-BO processed with **e** DIO, and **f** BN.


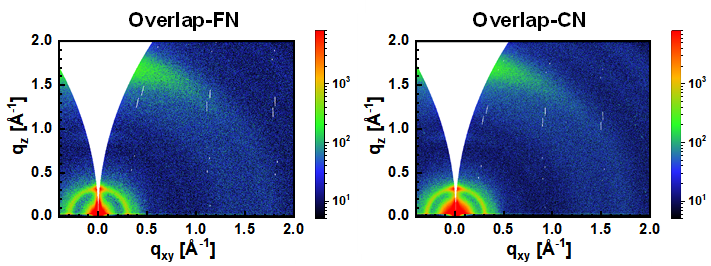


**Fig. S34**  2D-GIWAXS patterns of the PBDB-T-2F:BTP-BO-4Cl films processed from Overlap-FN, and Overlap-CN.

**Table S9** Calculated Flory-Huggins interaction parameter

| Additive | δ_D_ | δ_P_ | δ_H_ | Molar Volume  (cm^3^ /mol) | χ_PBDB-T-2F-Solvenr_ | χ_BTP-BO-4Cl-Solvenr_ | Boiling point  (℃) |
| --- | --- | --- | --- | --- | --- | --- | --- |
| FN | 19.5 | 5.5 | 3.0 | 129.1 | 0.23 | 0.41 | 215 |
| CN | 19.9 | 4.9 | 2.5 | 136.2 | 0.27 | 0.54 | 260 |


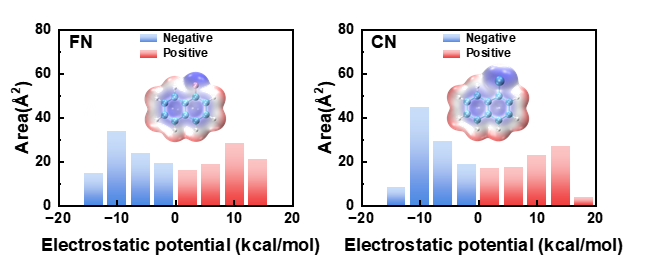


**Fig. S35** Surface Area of FN, and CN molecule within Different Electrostatic Potential Intervals [S3-S10].

**
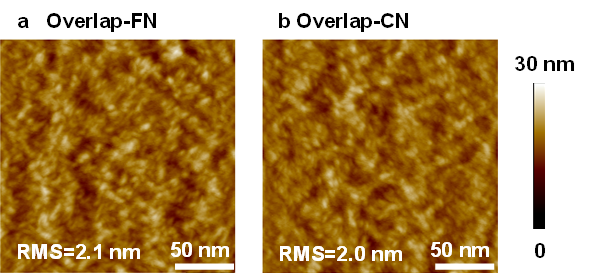
**

**Fig. S36** AFM height images of the blend films inkjet-printed from **a** FN, and **b** CN.

**Table S10** Performance parameters of conventional-structure OSCs with active layers fabricated from inkjet printing with different additive, under AM 1.5 G 100 mW/cm^2^ illumination

|  | Condition | *V*_OC_  (V) | *J*_SC_  (mA/cm^2^) | FF  (%) | PCE  (%) |
| --- | --- | --- | --- | --- | --- |
| PBDB-T-2F:  BTP-BO-4Cl | Overlap | 0.822 | 25.91 | 69.56 | 14.81 |
|  |  | 0.824±0.002 | 25.07±0.68 | 70.24±1.62 | 14.51±0.27 |
|  | Overlap-BN | 0.836 | 27.10 | 72.85 | 16.50 |
|  |  | 0.835±0.001 | 26.53±0.35 | 72.95±1.12 | 16.16±0.35 |
| PBDB-T-2F:BTP-BO-4Cl:PC_61_BM | Overlap | 0.836 | 26.79 | 72.19 | 16.16 |
|  |  | 0.834±0.003 | 26.54±0.37 | 71.86±1.64 | 15.90±0.22 |
|  | Overlap-BN | 0.843 | 27.72 | 75.22 | 17.57 |
|  |  | 0.839±0.004 | 27.72±0.18 | 73.99±0.85 | 17.20±0.24 |


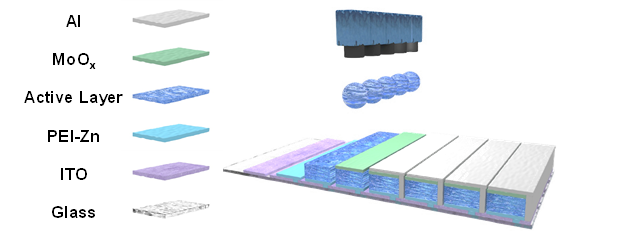


**Fig. S37** The Schematic diagram of the OSC module, illustrating the series-connected sub-cells and layer configuration: ITO/ETL/active layer/HTL/Al.

**Table S11** Photovoltaic parameters of inkjet printed mudules, under AM 1.5 G 100 mW/cm^2^ illumination

| Condition | *V*_OC_  (V) | *J*_SC_  (mA/cm^2^) | FF  (%) | PCE  (%) |
| --- | --- | --- | --- | --- |
| module | 7.048 | 2.67 | 69.29 | 13.04 |
|  | 6.873±0.257 | 2.67±0.02 | 64.53±3.79 | 11.92±0.95 |

**Supplementary References**

1. F. Urbach. The long-wavelength edge of photographic sensitivity and of the electronic absorption of solids. Phys. Rev. **92**(5), 1324-1324 (1953). <https://doi.org/https://doi.org/10.1103/PhysRev.92.1324>
2. J. Wu, J. Luke, H. K. H. Lee, P. Shakya Tuladhar, H. Cha, et al. Tail state limited photocurrent collection of thick photoactive layers in organic solar cells. Nat. Commun. **10**(1), 5159 (2019). <https://doi.org/https://doi.org/10.1038/s41467-019-12951-7>
3. 3. Y. Li, Z. Jia, P. Huang, C. Gao, Y. Wang, et al. Simultaneously improving the efficiencies of organic photovoltaic devices and modules by finely manipulating the aggregation behaviors of y-series molecules. Energy Environ. Sci. **18**(1), 256-263 (2025). <https://doi.org/https://doi.org/10.1039/D4EE04378B>
4. L. Ma, H. Yao, J. Wang, Y. Xu, M. Gao, et al. Impact of electrostatic interaction on bulk morphology in efficient donor–acceptor photovoltaic blends. Angew. Chem. Int. Ed. **60**(29), 15988-15994 (2021). <https://doi.org/https://doi.org/10.1002/anie.202102622>
5. M. J. Frisch, G. W. Trucks, H. B. Schlegel, G. E. Scuseria, M. A. Robb, et al. Gaussian 16 rev. C.03. (2016)
6. T. Lu, F. Chen. Multiwfn: A multifunctional wavefunction analyzer. J. Comput. Chem. **33**(5), 580-592 (2012). <https://doi.org/https://doi.org/10.1002/jcc.22885>
7. T. Lu. A comprehensive electron wavefunction analysis toolbox for chemists, multiwfn. J. Phys. Chem. Lett. **161**(8), 082503 (2024). <https://doi.org/https://doi.org/10.1063/5.0216272>
8. W. Humphrey, A. Dalke, K. Schulten. Vmd: Visual molecular dynamics. J. Mol. Graph. **14**(1), 33-38 (1996). <https://doi.org/https://doi.org/10.1016/0263-7855(96)00018-5>
9. S. Manzetti, T. Lu. The geometry and electronic structure of aristolochic acid: Possible implications for a frozen resonance. J. Phys. Org. Chem. **26**(6), 473-483 (2013). <https://doi.org/https://doi.org/10.1002/poc.3111>
10. T. Lu, S. Manzetti. Wavefunction and reactivity study of benzo[a]pyrene diol epoxide and its enantiomeric forms. Struct. Chem. **25**(5), 1521-1533 (2014). <https://doi.org/https://doi.org/10.1007/s11224-014-0430-6>
